# Australian guideline on prevention of foot ulceration: part of the 2021 Australian evidence-based guidelines for diabetes-related foot disease

**DOI:** 10.1186/s13047-022-00534-7

**Published:** 2022-07-06

**Authors:** Michelle R. Kaminski, Jonathan Golledge, Joel W. J. Lasschuit, Karl-Heinz Schott, James Charles, Jane Cheney, Anita Raspovic, on behalf of the Australian Diabetes-related Foot Disease Guidelines & Pathways Project

**Affiliations:** 1 Discipline of Podiatry, School of Allied Health, Human Services and Sport, La Trobe University, Melbourne, Victoria Australia; 2 Department of Podiatry, St Vincent’s Hospital Melbourne, Melbourne, Victoria Australia; 3 Queensland Research Centre for Peripheral Vascular Disease, College of Medicine and Dentistry, James Cook University, Townsville, Queensland Australia; 4 The Department of Vascular and Endovascular Surgery, The Townsville University Hospital, Townsville, Queensland Australia; 5 Department of Endocrinology and Diabetes, St Vincent’s Hospital, Sydney, New South Wales Australia; 6 Healthy Ageing, Garvan Institute of Medical Research, Sydney, New South Wales Australia; 7 Faculty of Medicine, University of New South Wales, Sydney, New South Wales Australia; 8 Southern Cross University School of Health and Human Sciences / Pedorthics, Gold Coast, Queensland Australia; 9 First Peoples Health Unit, Health Group, Griffith University, Gold Coast, Queensland Australia; 10 Diabetes Victoria, Melbourne, Victoria Australia; 11 Diabetes Feet Australia, Brisbane, Queensland Australia; 12 Australian Diabetes Society, Sydney, New South Wales Australia

**Keywords:** Diabetes-related foot ulceration, Diabetes-related foot disease, Education, Foot self-care, Foot ulcer, Footwear, Guideline, Prevention, Surgery

## Abstract

**Background:**

There are no current Australian guidelines on the prevention of diabetes-related foot ulceration (DFU). A national expert panel aimed to systematically identify and adapt suitable international guidelines to the Australian context to create new Australian evidence-based guidelines on prevention of first-ever and/or recurrent DFU. These guidelines will include for the first-time considerations for rural and remote, and Aboriginal and Torres Strait Islander peoples.

**Methods:**

The National Health and Medical Research Council procedures were followed to adapt suitable international guidelines on DFU prevention to the Australian health context. This included a search of public databases after which the International Working Group on the Diabetic Foot (IWGDF) prevention guideline was deemed the most appropriate for adaptation. The 16 IWGDF prevention recommendations were assessed using the ADAPTE and GRADE systems to decide if they should be adopted, adapted or excluded for the new Australian guideline. The quality of evidence and strength of recommendation ratings were re-evaluated with reference to the Australian context. This guideline underwent public consultation, further revision, and approval by national peak bodies.

**Results:**

Of the 16 original IWGDF prevention recommendations, nine were adopted, six were adapted and one was excluded. It is recommended that all people at increased risk of DFU are assessed at intervals corresponding to the IWGDF risk ratings. For those at increased risk, structured education about appropriate foot protection, inspection, footwear, weight-bearing activities, and foot self-care is recommended. Prescription of orthotic interventions and/or medical grade footwear, providing integrated foot care, and self-monitoring of foot skin temperatures (contingent on validated, user-friendly and affordable systems becoming available in Australia) may also assist in preventing DFU. If the above recommended non-surgical treatment fails, the use of various surgical interventions for the prevention of DFU can be considered.

**Conclusions:**

This new Australian evidence-based guideline on prevention of DFU, endorsed by 10 national peak bodies, provides specific recommendations for relevant health professionals and consumers in the Australian context to prevent DFU. Following these recommendations should achieve better DFU prevention outcomes in Australia.

## Background

Diabetes-related foot ulceration (DFU) is recognised as a leading cause of hospital admission and amputations worldwide [1–4]. DFU also contributes to high rates of morbidity and mortality, which poses a major burden to the health-related quality of life of patients and has substantial economic consequences [1, 5–7]. The lifetime incidence of DFU is between 19 to 34%, with an annual incidence of around 2% [1, 8]. DFU recurrence is also very common, with approximately 40% of ulcers recurring within 1 year and 65% within 3 years [1, 8].

In Australia, it is estimated that 50,000 people are living with DFU, while 300,000 people are considered at-risk [4–6, 9]. Each year, Australia has approximately 28,000 hospital admissions, 4500 amputations, 1700 deaths, and $AU1.6 billion in health care expenditure attributable to DFU [4, 10–12]. Aboriginal and Torres Strait Islander peoples have disproportionately high rates of foot-related complications, with a 3 to 6-fold increased likelihood of developing DFU and requiring amputation [5, 13, 14]. Prevention is central to reducing the very high national DFU burden and addressing the Closing the Gap in Partnership agreement to help Aboriginal and Torres Strait Islander peoples enjoy long and healthy lives [15].

Key risk factors contributing to the development of DFU include peripheral neuropathy, peripheral artery disease (PAD) and foot deformity [8, 16, 17]. Empirical evidence has shown that history of foot ulceration, amputation and/or end-stage renal disease (ESRD) further increase the risk [1, 8, 16, 17]. For those without risk factors, the incidence of DFU is very low [8]. Hence, prevention strategies should be targeted to people considered at increased risk (at-risk) for DFU [8]. Within existing studies and clinical practice, there are a variety of interventions available for the prevention of DFU, and for the treatment of modifiable risk factors in those at-risk [18, 19]. These prevention interventions include examining and inspecting the feet, structured education about foot self-care and management principles, early treatment of pre-ulcerative signs or injuries, surgical interventions (particularly to prevent ulcer recurrence), and the provision of integrated foot care [8, 18, 19].

Interventions aimed at the prevention of DFU have been found to have contrasting benefits and risks [18, 19], varying levels of evidence to support their benefits and risks [18, 19], and global differences in their feasibility and clinical uptake [20–22]. In order to interpret the balance between the benefits and risks, the quality of the supporting evidence, and the acceptability and feasibility of these interventions, evidence-based prevention guidelines have been developed to guide optimal care [8, 23].

The 2011 Australian evidence-based guidelines for the treatment of people with diabetes-related foot disease (DFD) are currently outdated [22, 23], which may be reflective of the high costs (~$AU1 million) associated with the development of new high-quality guidelines [24]. There is now a compelling need for the Australian DFD Guidelines to be updated to provide contemporary evidence-based recommendations to health professionals to help prevent the large national DFU burden [22]. A more cost-effective, yet still robust, alternative to the creation of new guidelines from scratch, is to adapt existing international guidelines; following rigorous assessment by experts in the field to ensure that they meet a high-quality standard [25]. In recent years, there have been several international evidence-based DFD guidelines published [8, 26–28]. Given the uncertainty and unlikelihood of securing substantial funding for the development of new Australian DFD Guidelines in the foreseeable future, the aim was to systematically identify and adapt suitable international guidelines to the Australian context. This paper presents the new Australian evidence-based guidelines on prevention of first-ever and/or recurrent DFU.

## Methods

### Key steps

The methodology for this guideline has been described in detail in an accompanying guidelines development paper authored by the Australian DFD Guidelines working group [29]. The National Health and Medical Research Council (NHMRC) procedures for adapting source guidelines were followed [25, 30, 31], using eight overarching steps: (i) defining the scope (population and problem); (ii) identifying potential source guidelines; (iii) assessing the suitability of source guidelines; (iv) assessing and deciding which source guideline recommendations to adopt, adapt or exclude in the new context; (v) drafting new recommendations and rationale for the context; (vi) collating recommendations and rationale into new guidelines; (vii) developing clinical pathway(s) to aide implementation; and (viii) consultation and endorsement of the final guidelines [29].

The development paper reports the findings of the initial three steps, including that the 2019 International Working Group on the Diabetic Foot (IWGDF) Guidelines [8] were identified and assessed as suitable international source guidelines to adapt for this new Australian guideline [29]. The subsequent steps are the subject of this manuscript and are outlined below.

### Prevention guideline panel

A national expert panel (‘the authors’) was established by the Australian DFD Guidelines working group to develop this prevention guideline, including recognised multi-disciplinary (inter) national clinical or research experts in the prevention of DFU, along with consumer, end-user and Aboriginal and Torres Strait Islander DFD experts [29]. The panel was provided all prevention recommendations (and all supporting rationale and evidence) from the IWGDF guidelines and systematic reviews [8, 18, 19] to consider as the basis for developing this guideline [29].

### Population of interest

The IWGDF identified the population of interest for their systematic reviews [18, 19] and subsequent guideline [8] as people at-risk of DFU (IWGDF risk stratification system: risk 1 [low], risk 2 [moderate], risk 3 [high]), defined as “people with diabetes mellitus and peripheral neuropathy”, including “people with or without foot deformities, PAD or lower- extremity amputation, and both people in remission from foot ulceration (i.e. foot ulcer history) and those with no foot ulcer history” (pp. 3) [18, 19].

### Initial screening with ADAPTE

Clinical and research panel members were assigned into pairs to independently screen each IWGDF prevention recommendation (and rationale) for their quality of evidence, strength of recommendation and the acceptability and applicability in the Australian context, using a customised 7-item ADAPTE evaluation form [29, 31].

The panel rated the *quality of evidence* in alignment with the Grading of Recommendations, Assessment, Development and Evaluations (GRADE) system as: high, if the panel was very confident that the findings were from studies reporting consistent effects with low risk of bias and further research was unlikely to change that confidence; moderate, if moderate confidence in the consistency of effects or risk of bias and further research was likely to impact that confidence; low, if limited confidence in the risk of bias or had inconsistency of effects and further research was very likely to impact confidence; and very low, if very little confidence in the available supporting evidence [30, 32, 33].

The panel also rated the *strength of recommendation* based on the GRADE system by weighing up the balance of effects, quality of evidence, values, applicability and acceptability [32, 33] in the Australian context [29] as: strong, if there was clearly a moderate-to-large difference in the balance of effects between the intervention compared with the control; and weak, if there was an uncertain and/or mild-to-moderate difference [32, 33]. Any disagreements between the two panel members on any ratings were discussed until consensus was reached. If consensus was not possible, a third member was involved in the adjudication.

Finally, the full panel met to discuss and gain consensus on all item ratings for all recommendations. Any recommendations in which the panel unanimously agreed with all items relating to the quality of evidence and strength of recommendation made by IWGDF, and acceptability and applicability in the Australian context, were adopted. Whereas any recommendations where the panel did not agree or were unsure on any item progressed to full assessment [29, 31].

### Full assessment with GRADE evidence to decision

Recommendations requiring full assessment were assessed using a customised GRADE Evidence to Decision (EtD) tool [29, 30, 32, 33]. This involved one panel member extracting and populating the EtD tool with all relevant supporting evidence text for the recommendation from the IWGDF prevention guideline and systematic reviews [8, 18, 19]. Eight important EtD criteria were specifically populated: the problem, desirable effects, undesirable effects, quality (or certainty) of evidence, values (of importance of outcomes), balance of effects, acceptability and applicability [29, 30, 32, 33]. Once populated, the EtD tool was checked by a second member for accuracy and any disagreements were discussed until a consensus was reached. This assessment involved the member(s) reading all populated text, adding any additional Australian literature or expert opinion considerations not included in the extracted IWGDF text, and making judgements for each criterion. The panel met to discuss and gain consensus on their summary judgements for the eight criteria [30, 32, 33] and compared their judgements with that of the IWGDF judgements [29, 30].

### Decision to adopt, adapt or exclude

Based on the level of agreement between the panel and IWGDF summary judgements, the panel then made a consensus decision on adopting, adapting or excluding the recommendation concerned for the Australian context [29, 30]. These decisions were defined as follows: adopted, if there were no substantial differences between the panel and IWGDF summary judgements; adapted, if there were substantial differences; and excluded, if there were substantial differences and/or the panel concluded the recommendation was not acceptable or applicable in Australia [29, 30]. Any disagreements within the panel were discussed until consensus was reached or, if that was not possible, by discussing with the Australian DFD Guideline working group until consensus was reached.

The panel then re-wrote any adapted recommendation to be clear, specific, and unambiguous as per the GRADE system [34, 35]. For each recommendation, the panel then drafted decision rationale, summary justifications for their judgements, detailed justifications for the important EtD criteria (if the recommendation was fully assessed), and considerations for implementation, special subgroups (including for geographically remote and Aboriginal and Torres Strait Islander populations), monitoring and future research priorities [30, 32, 33] in the Australian context [29]. The panel collated all recommendations (and rationale) into a consultation draft manuscript for the Australian evidence-based prevention guideline ready for public consultation [29].

### Clinical pathway development

Finalised recommendations were used to develop a DFU prevention clinical pathway [29]. The pathway aimed to optimise the implementation of prevention recommendations by the multiple health professionals and disciplines caring for Australians with DFU in secondary and tertiary health care settings in Australia. The pathway development methodology followed the 10-step process for developing and implementing clinical pathways as recommended by Flores et al. [36] and has also been outlined in detail in the accompanying Guideline development paper [29].

### Public consultation and peak body endorsement

The consultation draft manuscript of the prevention guideline underwent a formal six-week public consultation period using a 23-item customised consultation survey from ADAPTE [29, 31]. All relevant survey and written feedback data from the consultation period were collated, analysed and the manuscript was revised accordingly by the authors [29, 31]. Finally, the authors sought endorsement from the Australian DFD Guidelines working group and other relevant peak national bodies for the final guideline to be released [29]. The results and recommendations in this guideline should be read in conjunction with the respective source documents from the IWGDF Prevention Working Group, where full descriptions of the findings and rationale are provided [8, 18, 19].

## Results

**Fig. 1:**
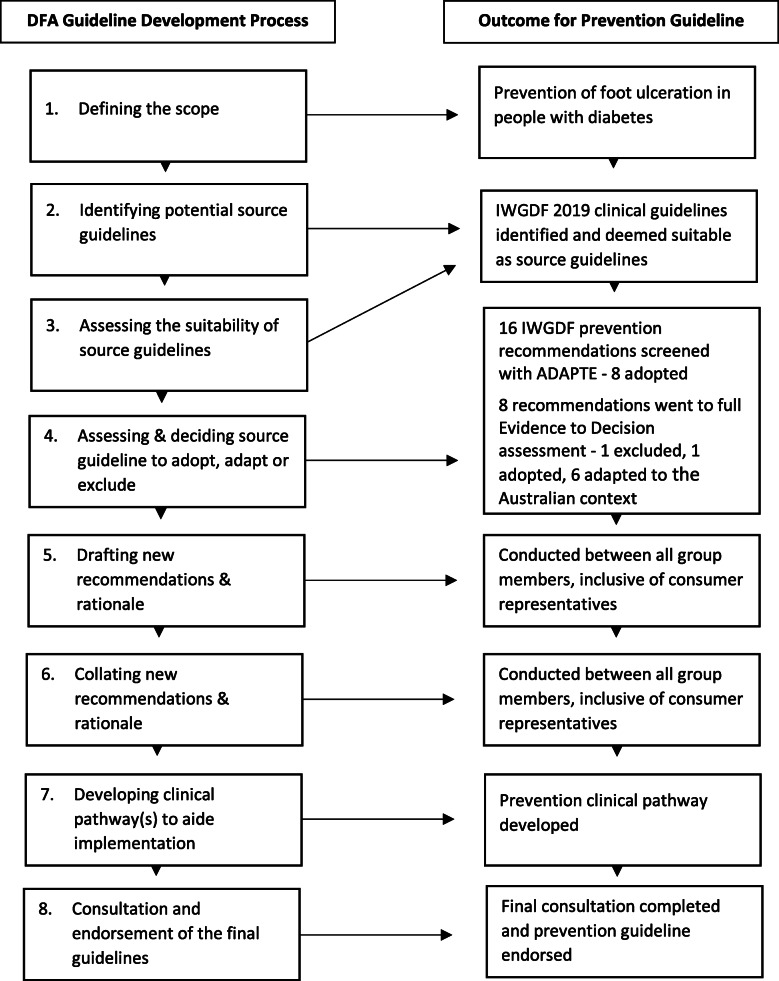
Application of the DFD guideline Adapting Process to the Prevention Guideline

Figure 1 displays a diagrammatic summary of the guideline development process and key outcomes. Table 1 shows that after screening, eight recommendations required further full assessment and eight were adopted without further assessment. Table 2 shows that of the eight recommendations that underwent full assessment, one was adopted, one was excluded and six were adapted in order to be considered acceptable and applicable in the Australian context. The main reasons for adapting, included two recommendations that had the quality of evidence rating downgraded, three that had the population or implementation requirements clarified (wordings of recommendations were restructured in an attempt to retain the same meaning but improve clarity) and one that had the intervention modified. The reasons for excluding (Recommendation 14 of the IWGDF Prevention Guideline) were due to the panel having substantial differences in judgements to the IWGDF for the desirable effects, balance of effects, and the quality of evidence, resulting in the panel concluding that the recommendation was not acceptable in the Australian context. Table 3 summarises the wording differences between each of the original 16 IWGDF recommendations and the new 15 Australian recommendations. Overall, nine were adopted (Recommendations 3–5, 7–10, 13 and 16), six were adapted (Recommendations 1–2, 6, 11–12 and 15) and one was excluded (Recommendation 14).

**Table 1:**
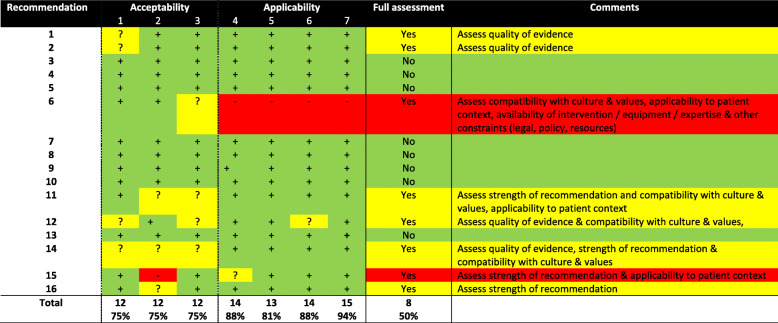
Summary of screening ratings for acceptability and applicability in the Australian context for all IWGDF prevention recommendations (ADAPTE ratings)

Note: +, yes item is met; −, no item is not met; ?, unsure if item is met

**Table 2:** Summary of final panel judgements compared with IWGDF judgements for all IWGDF prevention recommendations

No.	Problem	Desirable effects	Undesirable effects	Quality of evidence	Values	Balance of effects	Acceptability	Applicability/ feasibility	Decision	Comment
**1**	+ Yes	+ Moderate	+ Trivial	- Low	? Probably no important uncertainty	+ Favours the intervention	+ Yes	+ Yes	Adapt	Adapted QoE
**2**	+ Yes	+ Moderate	+ Trivial	- Low	? Probably no important uncertainty	+ Favours the intervention	+ Yes	+ Yes	Adapt	Adapted QoE
**3**	=	=	=	=	=	=	=	=	Adopt	Adopted in ADAPTE screening
**4**	=	=	=	=	=	=	=	=	Adopt	Adopted in ADAPTE screening
**5**	=	=	=	=	=	=	=	=	Adopt	Adopted in ADAPTE screening
**6**	+ Yes	+ Moderate	- Moderate	+ Moderate	+ Possibly important uncertainty	? Probably favours the intervention	? Probably yes	- No	Adapt	Adapted wording to reflect that the technology is not yet available in Australia
**7**	=	=	=	=	=	=	=	=	Adopt	Adopted in ADAPTE screening
**8**	=	=	=	=	=	=	=	=	Adopt	Adopted in ADAPTE screening
**9**	=	=	=	=	=	=	=	=	Adopt	Adopted in ADAPTE screening
**10**	=	=	=	=	=	=	=	=	Adopt	Adopted in ADAPTE screening
**11**	+ Yes	+ Moderate	+ Small	+ Low	+ Probably no important uncertainty	+ Probably favours the intervention	+ Varies	+ Varies	Adapt	Adapted wording to retain meaning, but improve clarity
**12**	+ Yes	+ Moderate	+ Moderate	+ Low	+ Probably no important uncertainty	+ Probably favours the intervention	+ Probably yes	+ Probably yes	Adapt	Adapted wording to retain meaning, but improve clarity
**13**	=	=	=	=	=	=	=	=	Adopt	Adopted in ADAPTE screening
**14**	+ Yes	- Don’t know	+ Trivial	- Very low	+ No important uncertainty	- Does not favour either the intervention or comparison	+ Probably yes	+ Yes	Exclude	Excluded due to low-quality supporting evidence
**15**	+ Yes	+ Small	? Small	+ Low	+ Probably no important uncertainty	? Probably favours the intervention	+ Probably yes	+ Probably yes	Adapt	Adapted intervention
**16**	+ Yes	+ Large	+ Small	+ Low	+ Probably no important uncertainty	+ Probably favours the intervention	+ Yes	+ Probably yes	Adopt	Adopted in EtD assessment

Note: +, panel agreed with original IWGDF judgement; -, panel disagreed with original IWGDF judgement; ?, panel unsure if agreed with original IWGDF judgement due to lack of IWGDF information on judgement; =, panel agreed with original IWGDF judgements during screening (see Table 1); *QoE* Quality of evidence

**Table 3:** Summary of the original IWGDF recommendation compared with the new Australian guideline recommendations for prevention

No.	Original IWGDF Recommendation	Decision	No.	New Australian Recommendation
**1**	Examine a person with diabetes at very low risk of foot ulceration (IWGDF risk 0) annually for signs or symptoms of loss of protective sensation and peripheral artery disease, to determine if they are at increased risk for foot ulceration. (GRADE recommendation: Strong; Quality of evidence: High)	Adapt	**1**	Examine a person with diabetes at very low risk of foot ulceration (IWGDF risk 0) annually for signs or symptoms of loss of protective sensation and peripheral artery disease, to determine if they are at increased risk for foot ulceration. (GRADE strength of recommendation: Strong; Quality of evidence: Low)
**2**	Screen a person with diabetes at risk of foot ulceration (IWGDF risk 1-3) for: a history of foot ulceration or lower-extremity amputation; diagnosis of end-stage renal disease; presence or progression of foot deformity; limited joint mobility; abundant callus; and any pre-ulcerative sign on the foot. Repeat this screening once every 6-12 months for those classified as IWGDF risk 1, once every 3-6 months for IWGDF risk 2, and once every 1-3 months for IWGDF risk 3. (Strong; High)	Adapt	**2**	Screen a person with diabetes at risk of foot ulceration (IWGDF risk 1-3) for: a history of foot ulceration or lower-extremity amputation; diagnosis of end-stage renal disease; presence or progression of foot deformity; limited joint mobility; abundant callus; and any pre-ulcerative sign on the foot. Repeat this screening once every 6-12 months for those classified as IWGDF risk 1, once every 3-6 months for IWGDF risk 2, and once every 1-3 months for IWGDF risk 3. (Strong; Low)
**3**	Instruct a person with diabetes who is at risk of foot ulceration (IWGDF risk 1-3) to protect their feet by not walking barefoot, in socks without shoes, or in thin-soled slippers, whether indoors or outdoors. (Strong; Low)	Adopt	**3**	As stated in original IWGDF recommendation
**4**	Instruct, and after that encourage and remind, a person with diabetes who is at risk of foot ulceration (IWGDF risk 1-3) to: inspect daily the entire surface of both feet and the inside of the shoes that will be worn; wash the feet daily (with careful drying, particularly between the toes); use emollients to lubricate dry skin; cut toe nails straight across; and, avoid using chemical agents or plasters or any other technique to remove callus or corns. (Strong; Low)	Adopt	**4**	As stated in original IWGDF recommendation
**5**	Provide structured education to a person with diabetes who is at risk of foot ulceration (IWGDF risk 1-3) about appropriate foot self-care for preventing a foot ulcer. (Strong; Low)	Adopt	**5**	As stated in original IWGDF recommendation
**6**	Consider instructing a person with diabetes who is at moderate or high risk of foot ulceration (IWGDF risk 2-3) to self-monitor foot skin temperatures once per day to identify any early signs of foot inflammation and help prevent a first or recurrent plantar foot ulcer. If the temperature difference is above-threshold between similar regions in the two feet on two consecutive days, instruct the patient to reduce ambulatory activity and consult an adequately trained health care professional for further diagnosis and treatment. (Weak; Moderate)	Adapt	**6**	Consider instructing a person with diabetes who is at moderate or high risk of foot ulceration (IWGDF risk 2-3) to self-monitor foot skin temperatures once per day to identify any early signs of foot inflammation and help prevent a first or recurrent plantar foot ulcer. The implementation of this recommendation is contingent on validated, user-friendly and affordable systems becoming approved and available in Australia. If the temperature difference is above-threshold between similar regions in the two feet on two consecutive days, instruct the patient to reduce ambulatory activity and consult an adequately trained health care professional for further diagnosis and treatment. (Weak; Moderate)
**7**	Instruct a person with diabetes who is at moderate risk for foot ulceration (IWGDF risk 2) or who has healed from a non-plantar foot ulcer (IWGDF risk 3) to wear therapeutic footwear that accommodates the shape of the feet and that fits properly, to reduce plantar pressure and help prevent a foot ulcer. When a foot deformity or a pre-ulcerative sign is present, consider prescribing custom-made footwear, custom-made insoles, or toe orthoses. (Strong; Low)	Adopt	**7**	As stated in original IWGDF recommendation, except ‘therapeutic footwear’ has been replaced with ‘medical grade footwear’ and ‘custom-made insoles’ has been replaced with ‘custom-made foot orthoses’, so that the terminology was applicable to the Australian context. Instruct a person with diabetes who is at moderate risk for foot ulceration (IWGDF risk 2) or who has healed from a non-plantar foot ulcer (IWGDF risk 3) to wear medical grade footwear that accommodates the shape of the feet and that fits properly, to reduce plantar pressure and help prevent a foot ulcer. When a foot deformity or a pre-ulcerative sign is present, consider prescribing custom-made footwear, custom-made foot orthoses, or toe orthoses. (Strong; Low)
**8**	Consider prescribing orthotic interventions, such as toe silicone or (semi-)rigid orthotic devices, to help reduce abundant callus in a person with diabetes who is at risk for foot ulceration (IWGDF risk 1-3). (Weak; Low)	Adopt	**8**	As stated in original IWGDF recommendation
**9**	In a person with diabetes who has a healed plantar foot ulcer (IWGDF risk 3), prescribe therapeutic footwear that has a demonstrated plantar pressure relieving effect during walking, to help prevent a recurrent plantar foot ulcer; furthermore, encourage the patient to consistently wear this footwear. (Strong; Moderate).	Adopt	**9**	As stated in original IWGDF recommendation, except ‘therapeutic footwear’ has been replaced with ‘medical grade footwear’, so that the terminology was applicable to the Australian context. In a person with diabetes who has a healed plantar foot ulcer (IWGDF risk 3), prescribe medical grade footwear that has a demonstrated plantar pressure relieving effect during walking, to help prevent a recurrent plantar foot ulcer; furthermore, encourage the patient to consistently wear this footwear. (Strong; Moderate).
**10**	Treat any pre-ulcerative sign or abundant callus on the foot, ingrown toe nail, and fungal infection on the foot, to help prevent a foot ulcer in a person with diabetes who is at risk of foot ulceration (IWGDF risk 1-3). (Strong; Low)	Adopt	**10**	As stated in original IWGDF recommendation
**11**	In a person with diabetes and abundant callus or an ulcer on the apex or distal part of a non-rigid hammertoe that has failed to heal with non-surgical treatment, consider digital flexor tendon tenotomy for preventing a first foot ulcer or recurrent foot ulcer once the active ulcer has healed (Weak; Low).	Adapt	**11**	In a person with diabetes and abundant callus consider digital flexor tendon tenotomy for preventing a first foot ulcer. Where there is an ulcer on the apex or distal part of a non-rigid hammertoe that has failed to heal with evidence-based non-surgical treatment, consider this procedure to help prevent future ulcer recurrence. (Weak; Low)
**12**	In a person with diabetes and a plantar forefoot ulcer that has failed to heal with non-surgical treatment, consider Achilles tendon lengthening, single or pan metatarsal head resection, metatarsophalangeal joint arthroplasty or osteotomy, to help prevent a recurrent plantar forefoot ulcer once the active ulcer has healed. (Weak; Low)	Adapt	**12**	In a person with diabetes and a plantar forefoot ulcer that has failed to heal with evidence-based non-surgical treatment, consider Achilles tendon lengthening, single or pan metatarsal head resection, metatarsophalangeal joint arthroplasty or osteotomy, to help prevent future ulcer recurrence. (Weak; Low)
**13**	We suggest not to use a nerve decompression procedure, in preference to accepted standards of good quality care, to help prevent a foot ulcer in a person with diabetes who is at moderate or high risk of foot ulceration (IWGDF risk 2-3) and who is experiencing neuropathic pain. (Weak; Low)	Adopt	**13**	As stated in original IWGDF recommendation
**14**	Consider advising a person with diabetes who is at low or moderate risk for foot ulceration (IWGDF risk 1 or 2) to perform foot and mobility-related exercises with the aim of reducing risk factors of ulceration, that is, decreasing peak pressure and increasing foot and ankle range of motion, and with the aim of improving neuropathy symptoms. (Weak; Moderate)	Exclude	–	Recommendation excluded from the Australian guideline
**15**	Consider communicating to a person with diabetes who is at low or moderate risk for foot ulceration (IWGDF risk 1 or 2) that a moderate increase in the level of walking-related weight-bearing activity (ie, an extra 1.000 steps/day) is likely to be safe. Advise this person to wear appropriate footwear when undertaking weight-bearing activities, and to frequently monitor the skin for pre-ulcerative signs or breakdown. (Weak; Low)	Adapt	**14**	Consider communicating to a person with diabetes who is at risk of foot ulceration (IWGDF risk 1-3) that any increase in weight-bearing activity should be gradual, ensuring appropriate footwear and/or prescribed offloading device(s) are worn, and that the skin is frequently monitored for pre-ulcerative signs or injury. (Weak; Low)
**16**	Provide integrated foot care for a person with diabetes who is at high risk of foot ulceration (IWGDF risk 3) to help prevent a recurrent foot ulcer. This integrated foot care includes professional foot care, adequate footwear and structured education about self-care. Repeat this foot care or re-evaluate the need for it once every one to three months, as necessary. (Strong; Low)	Adopt	**15**	As stated in original IWGDF recommendation

Note: Underlined wording indicates the specific adaptations to the original IWGDF recommendation

**Fig. 2:**
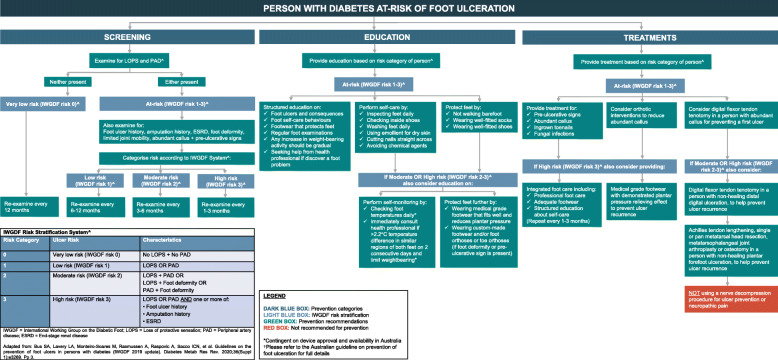
Australian clinical pathway to guide evidence-based prevention of foot ulcers in people at-risk of diabetes-related foot ulceration†

For each of the 15 Australian prevention recommendations, the question the recommendation addressed; the recommendation(s); the panel’s decision and rationale to adopt, adapt or exclude; a summary justification for the recommendation(s); and considerations for the Australian context (including for geographically remote and Aboriginal and Torres Strait Islander peoples) are outlined. As the panel agreed with the IWGDF for the majority of the summary judgements for EtD criteria across 15 (out of 16) of the original IWGDF recommendations, the differences in agreement to the IWGDF recommendations will be the focus of the results presented. The recommendations are displayed in order according to their prevention category: A. Identifying the at-risk foot; B. Regularly inspecting and examining the at-risk foot; C. Instructions on foot self-care; D. Providing structured education about foot self-care; E. Instructions about foot self-management; F. Ensuring routine wearing of appropriate footwear; G. Treatment of risk factors or pre-ulcerative signs on the foot; H. Surgical interventions; I. Foot-related exercises and weight-bearing activity; J. Integrated foot care. Figure 2 incorporates all 15 recommendations in a one-page Australian clinical pathway to guide evidence-based prevention of DFU. Finally, a glossary of terms used in the guideline which aligned with the IWGDF Guideline can be found at the end of this article.

Nineteen responses (13 individuals and six organisations) to the public consultation survey were received, with 18 completing the survey in its entirety. The collated public consultation responses are displayed in Table 4. No respondents (0%) disagreed with the statements that there was a need for a new prevention guideline, the methodology used for these guidelines was appropriate, the recommendations were clear, when applied the recommendations should produce more benefits than harms, and they would be comfortable if people with DFU received these recommendations. In implementing the prevention guideline, some respondents agreed that to apply the recommendations this may pose challenges around the need for reorganisation of services (50%), technical application (44%) and expense (22%). A large proportion of respondents (83%) agreed the guidelines are likely to be acceptable to people living with DFU. Overall, 78% of the respondents agreed (with none disagreeing) that the guideline should be approved as the new Australian prevention guideline. No respondents (0%) disagreed with the statements that the guideline would be supported by the majority of their colleagues and would encourage its use in practice. All de-identified feedback comments received during public consultation and the panel’s responses to each comment were collated and posted on the Diabetes Feet Australia website.

**Table 4:** Summary of public consultation survey responses (*n* = 19)

No.	Item	n	Strongly Agree	Agree	Neither Agree or Disagree	Disagree	Strongly Disagree
	**Background**						
1	You are involved with the care of patients for whom this draft Australian prevention guideline is relevant.	19	12 (63.2%)	4 (21.0%)	3 (15.8%)	0	0
2	There is a need for a new Australian prevention guideline in this population.	19	9 (47.4%)	9 (47.4%)	1 (5.2%)	0	0
3	The rationale for developing a new Australian prevention guideline on this topic is clear in this draft guideline.	19	12 (63.2%)	6 (31.6%)	1 (5.2%)	0	0
	**Methodology**						
4	I agree with the overall methodology used to develop this draft Australian prevention guideline.	19	7 (36.8%)	10 (52.6%)	2 (10.5%)	0	0
5	The search strategy used to identify international guidelines on which this draft Australian prevention guideline was based is relevant and complete	19	7 (36.8%)	10 (52.6%)	2 (10.5%)	0	0
6	The methods used to determine the suitability of identified international source guidelines upon which this draft Australian prevention guideline were based were robust.	19	8 (42.1%)	8 (42.1%)	3 (15.8%)	0	0
7	I agree with the methods used within this draft Australian prevention guideline to interpret the available evidence on this topic.	19	6 (31.6%)	11 (57.9%)	2 (10.5%)	0	0
8	The methods used to decide which recommendations to adopt, adapt or exclude for the Australian context were objective and transparent.	19	5 (26.3%)	12 (63.2%)	2 (10.2%)	0	0
	**Recommendations**						
9	The recommendations in this draft Australian prevention guideline are clear.	18	7 (38.9%)	10 (55.6%)	1 (5.6%)	0	0
10	I agree with the recommendations in this draft Australian prevention guideline as stated.	18	3 (16.7%)	13 (68.4%)	1 (5.6%)	1 (5.6%)	0
11	The recommendations are suitable for people living with diabetes-related foot disease.	18	4 (22.2%)	13 (68.4%)	1 (5.6%)	0	0
12	The recommendations are too rigid to apply for people living with diabetes-related foot disease.	18	1 (5.6%)	1 (5.6%)	4 (22.2%)	11 (61.1%)	1 (5.6%)
13	The recommendations reflect a more effective approach to improving patient outcomes than is current practice.	18	2 (11.1%)	7 (38.9%)	8 (44.4%)	1 (5.6%)	0
14	When applied, the recommendations should produce more benefits than harms for people living with diabetes-related foot disease.	18	8 (44.4%)	9 (50.0%)	1 (5.6%)	0	0
15	When applied, the recommendations should result in better use of resources than current practice allows.	18	6 (33.3%)	5 (27.8%)	6 (33.3%)	1 (5.6%)	0
16	I would feel comfortable if people living with diabetes-related foot disease received the care recommended in this draft Australian prevention guideline.	18	7 (38.9%)	9 (50.0%)	2 (11.1%)	0	0
	**Implementation of recommendations**						
17	To apply the draft Australian prevention guideline may require reorganisation of services/care.	18	3 (16.7%)	6 (33.3%)	7 (38.9%)	2 (11.1%)	0
18	To apply the draft Australian prevention guideline may be technically challenging.	18	0	8 (44.4%)	7 (38.9%)	2 (11.1%)	1 (5.6%)
19	The draft Australian prevention guideline may be too expensive to apply.	18	2 (11.1%)	2 (11.1%)	7 (38.9%)	7 (38.9%)	0
20	The draft Australian prevention guideline presents options that will likely be acceptable to people living with diabetes-related foot disease.	18	3 (16.7%)	12 (66.7%)	1 (5.6%)	2 (11.1%)	0
	**Final thoughts**						
21	This draft guideline should be approved as the new Australian prevention guideline.	18	6 (33.3%)	8 (44.4%)	4 (22.2%)	0	0
22	This draft Australian prevention guideline would be supported by the majority of my colleagues.	18	7 (38.9%)	8 (44.4%)	3 (16.7%)	0	0
23	If this draft guideline was to be approved as the new Australian prevention guideline, I would use or encourage their use in practice.	18	8 (44.4%)	8 (44.4%)	2 (11.1%)	0	0

Based on the collated public consultation feedback, the guideline was revised, approved by the panel and Australian DFD Guidelines working group, and endorsed as the new *Australian guideline on prevention of foot ulceration* by ten peak national bodies including the Australian Podiatry Association, Wounds Australia, Australian and New Zealand Society for Vascular Surgery, Australasian Society for Infectious Diseases, Australian Orthotic Prosthetic Association, Pedorthic Association of Australia, Australian Advanced Practicing Podiatrists - High Risk Foot Group, Australian Aboriginal and Torres Strait Islander Diabetes-related Foot Complications Program, Australian Diabetes Society, and Diabetes Feet Australia.

## Recommendations

A. IDENTIFYING THE AT-RISK FOOT

The IWGDF risk stratification system referred to throughout is detailed in Table 5.

**Q1. In people with diabetes, is structured annual screening for risk factors of foot ulceration, compared with less frequent or unstructured screening, effective for preventing a first-ever or recurrent DFU?**

**Table 5:** The IWGDF Risk Stratification System*

Risk Category	Ulcer Risk	Characteristics
0	Very low	No LOPS + No PAD
1	Low	LOPS OR PAD
2	Moderate	LOPS + PAD OR LOPS + Foot deformity OR PAD + Foot deformity
3	High	LOPS OR PAD AND one or more of the following: • history of a foot ulcer • a lower-extremity amputation (minor or major) • end-stage renal disease

Note: *IWGDF* International Working Group on the Diabetic Foot, *LOPS* Loss of protective sensation, *PAD* Peripheral artery disease

*Adapted from: Bus SA, Lavery LA, Monteiro-Soares M, Rasmussen A, Raspovic A, Sacco ICN, et al. Guidelines on the prevention of foot ulcers in persons with diabetes (IWGDF 2019 update). Diabetes Metab Res Rev. 2020;36(Suppl 1):e3269. Pp 3

### Recommendation 1

Examine a person with diabetes at very low risk of foot ulceration (IWGDF risk 0) annually for signs or symptoms of loss of protective sensation and peripheral artery disease, to determine if they are at increased risk for foot ulceration. (GRADE strength of recommendation: Strong; Quality of evidence: Low).

**Decision**: Adapt.

### Rationale

The panel decided to adapt this original IWGDF recommendation, based on having a differing judgement to the IWGDF on the quality of evidence rating. Therefore, we downgraded the quality of evidence from high to low (Table 2).

### Summary of justification to adapt

Although the panel agreed with the IWGDF that the strength of this recommendation is strong, we disagreed that the quality of evidence supporting this is high [8]. The reason for our divergent judgement is that in our assessment while evidence exists supporting loss of protective sensation and PAD as risk factors for foot ulceration [16], no direct evidence, or a very low quality of supporting evidence, was available affirming the degree to which screening for these risk factors translates into prevention of DFU [8]. Furthermore, in our expert opinion, there are several scenarios whereby detection of ulcer risk upon screening might not result in DFU prevention, such as non-adherence to ulcer prevention strategies or deterioration in ulcer risk status between screenings. The panel therefore deemed that further research is required before a quality of evidence rating above low can be considered for this recommendation.

Otherwise, on all other EtD criteria judgements that led to the strength of recommendation rating, the panel were closely aligned with the IWGDF. There was strong agreement that identification of foot ulcer risk was, on face value and in our expert opinion, highly important for appropriate and targeted DFU preventative treatment and most probably offered at least moderate additional desirable effects (benefit) compared with not examining for foot ulcer risk. The panel, including consumer representatives, acknowledged that fast and effective approaches to identifying DFU risk were also of great value to persons with diabetes, and essential to receiving the right support and care. Conversely, undesirable effects from the possibility of the individual sustaining harm from screening was considered very unlikely, or trivial at best compared to not screening, given its non-invasive, inexpensive, and fast administration. Thus, the balance of effects favoured screening for increased risk compared to not screening, based on the difference between at least moderate likely desirable effects and trivial likely undesirable effects.

The panel also agreed that costs on a societal level, for example, across large publicly funded health services, may be a challenge to address. Arguably, the monetary costs of screening, however, are probably significantly outweighed by its benefits, although empirical data to inform this debate is not currently available. Optimal time periods for re-screening also need to be determined when considering costs versus benefits. The panel agreed that yearly screenings were likely to be acceptable and feasible for most people with diabetes at very low risk of DFU. Thus, overall, we agree with the IWGDF that the strength of the recommendation is strong, based on a clear balance of effects, acceptability and feasibility for screening for increased risk compared to not screening.

B. REGULARLY INSPECTING AND EXAMINING THE AT-RISK FOOT

The IWGDF risk stratification system referred to throughout is detailed in Table 5.

**Q2. In people with diabetes at risk for foot ulceration, what are the risk factors that should be screened for, for preventing a first-ever or recurrent DFU?**

### Recommendation 2

Screen a person with diabetes at risk of foot ulceration (IWGDF risk 1–3) for: a history of foot ulceration or lower-extremity amputation; diagnosis of end-stage renal disease; presence or progression of foot deformity; limited joint mobility; abundant callus; and any pre-ulcerative sign on the foot. Repeat this screening once every 6–12 months for those classified as IWGDF risk 1, once every 3–6 months for IWGDF risk 2, and once every 1–3 months for IWGDF risk 3. (Strong; Low).

**Decision:** Adapt.

### Rationale

The panel decided to adapt this recommendation as we had differing judgements for the quality of evidence rating. Therefore, we downgraded the quality of evidence rating from high to low (Table 2).

### Summary of justification to adapt

As for recommendation 1, the panel agreed with the IWGDF that the strength of recommendation 2 is also strong, however disagreed that the quality of evidence is high with similar justification [8]. While the key factors which are predictive of re-ulceration have a high quality of supporting evidence [1, 16, 17, 37], the degree to which screening for these factors is effective for prevention of DFU, and the optimal intervals for screening, have no evidence to our knowledge, or a very low quality of supporting evidence. The panel therefore also deemed that further research is required before a collective quality of evidence rating above low can be considered for this recommendation as well.

Otherwise, again the panel agreed with all other EtD criteria judgements that led to a strong strength of recommendation, including supporting the judgement that timely and targeted screening of people at risk of DFU, for the aforementioned risk factors, is pertinent for good care and offered at least a moderate additional desirable effect compared to not screening. It makes good intuitive sense that more frequent screening may result in early identification of risk factors, earlier intervention and, based on expert opinion, better prognosis in people who are already at risk. We agree that customised preventative treatment following screening is likely to outweigh possible harms, if treatment is provided by a suitably trained health care professional following evidence-based practice. The panel note that such screening may be anxiety provoking for some people, however agreed that it conversely offers additional opportunity for education and psychological support that individuals may value in addressing fears around developing a DFU and thus trivial undesirable effects. Therefore, similarly to recommendation 1, the balance of effects favoured screening for these risk factors compared with not screening, and screening was deemed acceptable, inexpensive, and thus feasible to most individuals. Although, costs at a societal level may be challenging to assess with the available evidence. Optimal time periods for re-screening also need to be determined when considering costs versus benefits. Taken together, the panel agrees that the strength of recommendation is strong for foot screening according to recommendations 1 and 2.

### Considerations for the Australian context: recommendations 1 and 2

We have summarised suggestions for health professionals to consider for implementing screening in Table 6 (for recommendation 1) and Table 7 (for recommendation 2), and otherwise we refer readers to the IWGDF Prevention Guidelines [8] for full details on screening considerations (pp. 3–5). It is the panel’s view that these screening tests and protocols are already widely used in current practice and there are few additional considerations for translation into the Australian context. Given the geographical size and diversity of Australia, and the sometimes limited availability of health services or services with sufficient training (see glossary for definition), some individuals may not be able to access timely screening as recommended. For example, Aboriginal and Torres Strait Islander populations in rural and remote areas of Australia may not be able to access such screening routinely due to a lack of services or factors such as seasonal movement. Of note, screening should be performed by an adequately trained heath care professional (such as a general practitioner, podiatrist, diabetes educator), which may add an additional barrier to its availability. However, the panel considers that to adequately train health professionals to competently perform foot risk screening is not a complex activity and there are a number of training programs or tools available to address this activity (such as the Indigenous Diabetic Foot Program [49] and the Foot Forward Train the Trainer Program [50]). Care should be taken to monitor a person’s risk status over time and adjust the screening interval according to any changes in risk status (see Table 5). For example, risk status would be increased if foot complications occurred. Therefore, due to potential limited access, movement (e.g. cultural practices), greater severity of diabetes, and greater risk of complications of some Aboriginal and Torres Strait Islander people, health professionals may also consider opportunistic screening and/or more frequent screening (e.g. every 6 months).

C. INSTRUCTIONS ON FOOT SELF-CARE

**Table 6:** Summary of IWGDF screening suggestions for Recommendation 1*

**Foot screening:**
• Aims to identify those at risk of DFU. • Should specifically include screening for LOPS caused by peripheral neuropathy and for signs or symptoms of PAD. • Should be performed by an adequately trained health care professional (see glossary for definition) who is also aware of the evidence on screening validity and is competent to undertake further assessment / intervention and/or referral to a suitably trained health care practitioner to ensure individuals receive suitable care. • The examination should include (but is not limited to): - screening for LOPS with a 10-g Semmes Weinstein monofilament [26], or if unavailable, use of the Ipswich Touch Test [38] and screening of vibratory sensation with a tuning fork or biothesiometer/neurothesiometer, if the monofilament testing is negative; - screening for PAD as per the IWGDF Guidelines on PAD [39] and/or the Australian DFD Guidelines on PAD [40] by taking a cardiovascular history, palpating for foot pulses, obtaining pedal Doppler arterial waveforms and blood pressure measurements; - although evidence for a screening interval is non-existent, we recommend an annual screening for a person with diabetes in whom LOPS or PAD have not yet been identified; - further assessment / intervention and/or referral for follow-up if any areas of concern are identified.

Note: *DFD* Diabetes-related foot disease, *DFU* Diabetes-related foot ulceration, *IWGDF* International Working Group on the Diabetic Foot, *LOPS* Loss of protective sensation, *PAD* Peripheral artery disease

^*^Adapted from: Bus SA, Lavery LA, Monteiro-Soares M, Rasmussen A, Raspovic A, Sacco ICN, et al. Guidelines on the prevention of foot ulcers in persons with diabetes (IWGDF 2019 update). Diabetes Metab Res Rev. 2020;36(Suppl 1):e3269. Pp 4-5

**Table 7:** Summary of IWGDF screening suggestions for Recommendation 2*

**When a person with diabetes is identified as being at-risk of foot ulceration (i.e. IWGDF risk 1 to 3):**
• More extensive and more frequent foot examination is needed, as the ulcer risk is higher. • The examination should include (but is not limited to): - taking a detailed history of foot ulceration, lower-extremity amputation, and determining a diagnosis of end-stage renal disease; - physical examination of the foot for presence of deformities or progression thereof; abundant callus and pre-ulcerative signs, such as blisters, fissures and haemorrhage; and limited joint mobility; - history of other factors (suggested based more on expert opinion), including social isolation, poor access to health care, financial constraints, foot pain (with walking or at rest), and numbness or claudication; - examining the presence of ill-fitting, inadequate, or lack of footwear, abnormal skin colour, temperature or oedema; poor foot hygiene (e.g. improperly cut toenails, unwashed feet, superficial fungal infection, or unclean socks), physical limitations that may hinder foot self-care (e.g. visual acuity, obesity); and foot care knowledge are also suggested; • Any foot ulcer identified during screening should be treated according to the principles outlined in the suite of IWGDF Guidelines [26, 39, 41–44] and/or the Australian DFD Guidelines [40, 45–48].

Note: *DFD* Diabetes-related foot disease, *IWGDF* International Working Group on the Diabetic Foot

^*^Adapted from: Bus SA, Lavery LA, Monteiro-Soares M, Rasmussen A, Raspovic A, Sacco ICN, et al. Guidelines on the prevention of foot ulcers in persons with diabetes (IWGDF 2019 update). Diabetes Metab Res Rev. 2020;36(Suppl 1):e3269. Pp 4-5

‘Foot self-care’ and ‘foot self-management’ (see glossary for definitions) are two closely related interventions that both aim to reduce the risk of DFU and its associated complications. Foot self-care interventions (e.g. foot inspection, using emollients to lubricate dry skin, footwear inspection, etc) can be performed independently by the patient at home, whereas foot self-management involves more advanced assistive interventions, such as home monitoring systems (e.g. foot skin temperatures), lifestyle interventions, and telehealth [8, 51, 52]. The uptake of education and tasks relevant to foot self-care and foot self-management will be dependent on the individual’s unique physical and psychosocial circumstances and capacity to meet their particular requirements. Therefore, patients are encouraged to seek further support, or supports be arranged with the appropriate consents, if a patient is unable to perform these tasks themselves.

**Q3. In people with diabetes at risk for foot ulceration, is foot self-care compared to no self-care, effective for preventing a first-ever or recurrent DFU?**

### Recommendation 3

Instruct a person with diabetes who is at risk of foot ulceration (IWGDF risk 1–3) to protect their feet by not walking barefoot, in socks without shoes, or in thin-soled slippers, whether indoors or outdoors. (Strong; Low).

**Decision:** Adopt.

### Rationale

The panel adopted this recommendation as there was full agreement with the IWGDF regarding the strength of the recommendation and quality of evidence ratings and its applicability in the Australian context (Table 2).

### Summary of justification to adopt

The panel agreed with the IWGDF that there is low-quality supporting evidence for this recommendation. However, given that walking unprotected could be harmful and result in foot ulceration or external/mechanical trauma to the foot [8, 26, 53, 54], there was strong agreement with the IWGDF that education pertaining to the protection of the feet is a highly important DFU prevention strategy [8]. While some patients may prefer not to adhere to this recommendation, particularly when inside the home, the panel suggests that the benefits outweigh any potential harms or burden to the patient. On all other points of assessment for recommendation 3, the panel were closely aligned to the rationale of the IWGDF. The panel, including consumer representatives, agreed that education in how to protect the feet is likely to be acceptable and feasible for most people with diabetes.

Protecting the feet from high mechanical stress and external physical trauma is essential for reducing the risk of ulceration in a person with diabetes at risk of foot ulceration [8, 55]. This is also an important consideration in the Australian context; walking barefoot (e.g. on the beach) or with open type footwear is common, particularly in parts of Australia with hot climates. While this recommendation focuses on the protection of the feet both indoors or outdoors by not walking barefoot, in socks without shoes, or in thin-soled slippers, the panel agreed with the IWGDF that the use of any open type footwear increases the risk for direct damage to the skin by a foreign object [8, 55], but may also increase the risk of sunburn to the feet in the Australian context. While there is little empirical evidence to support the avoidance of open type of footwear in reducing the risk of ulceration, the panel suggests that closed-toe footwear is recommended as it protects the feet from mechanical impact, as well as reduces the risk of trauma and the collection of foreign objects. In exceptional circumstances (e.g. if the patient refuses to wear closed-toe footwear), sandals that can be properly fastened and have plantar pressure offloading ability that has been verified in each individual case, may be considered in preference to the patient walking barefoot, in socks, or in slip-on footwear. Although there is no evidence to support that wearing socks when in footwear reduces friction/shearing forces, based on expert opinion, the panel recommends that socks should be worn as this may reduce the risk of blistering, rubbing, or ulceration [55]. In addition, wearing clean socks when in shoes may also reduce the incidence of skin and nail infections (e.g. fungal infections) [56].

### Recommendation 4

Instruct, and after that encourage and remind, a person with diabetes who is at risk of foot ulceration (IWGDF risk 1–3) to: inspect daily the entire surface of both feet and the inside of the shoes that will be worn; wash the feet daily (with careful drying, particularly between the toes); use emollients to lubricate dry skin; cut toe nails straight across; and, avoid using chemical agents or plasters or any other technique to remove callus or corns. (Strong; Low).

**Decision:** Adopt.

### Rationale

The panel adopted this recommendation as there was full agreement with the IWGDF regarding the strength of the recommendation and quality of evidence ratings and its applicability in the Australian context (Table 2).

### Summary of justification to adopt

The panel agreed with the IWGDF that although there is low-quality supporting evidence for this recommendation, the strength of the recommendation should be considered ‘strong’ based on the balance of effects favouring foot self-care for the prevention of a first-ever or recurrent DFU (by detecting early signs of DFU and contributing to basic foot hygiene) [8]. On all other points of assessment for recommendation 4, the panel were closely aligned to the rationale of the IWGDF. The panel, including consumer representatives, agreed that education in performing good foot self-care practices is likely to be acceptable and feasible for most people with diabetes.

D. PROVIDING STRUCTURED EDUCATION ABOUT FOOT SELF-CARE

**Q4. In people with diabetes at risk of foot ulceration, is providing structured education about foot specific self-care compared to not providing it, effective for preventing a first-ever or recurrent DFU?**

### Recommendation 5

Provide structured education to a person with diabetes who is at risk of foot ulceration (IWGDF risk 1–3) about appropriate foot self-care for preventing a foot ulcer. (Strong; Low).

**Decision:** Adopt.

### Rationale

The panel adopted this recommendation as there was full agreement with the IWGDF regarding the strength of the recommendation and quality of evidence ratings and its applicability in the Australian context (Table 2).

### Summary of justification to adopt

When considering the balance of effects favouring structured foot self-care education over no education for the prevention of a first-ever or recurrent DFU, the panel were in agreement with the IWGDF that although there is low-quality supporting evidence for this recommendation, the strength of the recommendation should be considered ‘strong’ [8]. Despite education potentially resulting in a fear of complications for the patient, there was strong agreement with the IWGDF that structured education pertaining to: foot ulcers and their consequences; positive foot self-care behaviours; wearing protective footwear; undergoing regular foot checks; performing proper foot hygiene; and seeking professional help in a timely manner when a foot problem is discovered are all important DFU prevention strategies [8]. Providing structured education may also serve as a forum for patients to clarify any questions or uncertainties they have regarding their foot health management. On all other points of assessment for recommendation 5, the panel were closely aligned to the rationale of the IWGDF. Given the potential consequences and clinical sequelae of DFU, the panel and consumer representatives agreed that receiving structured education aimed at preventing DFU is likely to be acceptable and feasible for most people with diabetes at risk of ulceration.

### Considerations for the Australian context: recommendations 3, 4 and 5

Structured education on foot self-care practises is an essential component of foot ulcer prevention in an at-risk person with diabetes [8]. Specific examples of patient education include, but are not limited to, explaining the need for daily inspection of all surfaces of the feet including between the toes, ensuring the patient knows when and how to contact the appropriate health professional if signs of inflammation or pre-ulcerative signs are present or if there is a breach to the skin such as an ulcer, and specific foot practices such as using emollients to lubricate the skin (but not between the toes). Refer to the IWGDF Practical Guidelines [26] for further details. Furthermore, the education provided should be appropriate to the person’s culture, level of health literacy and preferred learning style (e.g. visual, verbal, written, illustrated).

From an Australian perspective, those living in geographically remote locations, where Aboriginal and Torres Strait Islander people account for a higher proportion of this population, may have limited availability of health services and adequately trained health professionals to provide such education. Likewise, these individuals may also have infrequent access or limited ability to attend for medical care to receive this foot care education; all of which may act as potential barriers for implementing these recommendations. However, national programs such as the ‘Foot Forward Train the Trainer Program’ [50] may aid in developing widespread competencies in foot screening, providing appropriate foot self-care education, and appropriate escalation of clinical care.

Performing foot self-care practises is particularly important for those living in rural or remote areas of Australia with hot climates; as this may precipitate perspiration and increased risk of blistering and/or ulceration. And similarly, for dry and dusty environments, people may need to wash their feet more regularly and check for any abrasions, sunburn, or injuries from foreign objects, particularly if people are wearing open type footwear or walking barefoot.

The panel suggest that special considerations may need to be made for the delivery of educational programs for those living in rural or remote areas of Australia. Telehealth services may play an important role in addressing this issue, however, further research into its effectiveness is required [57]. Other examples of delivery may include high-risk foot service teams visiting communities to facilitate education, drop-in foot clinics or education through other multimedia platforms. In both cases, equipment and resources would need to be made available to health care services and patients, which may not always be feasible. It is likely that some services may be better resourced than others to support such programs.

Health disparities between Aboriginal and Torres Strait Islander peoples and non-Indigenous Australians have been well documented [58–61]. Poorer health outcomes for Aboriginal and Torres Strait Islander populations are in part due to a higher prevalence of chronic diseases such as diabetes, but is further accentuated by geographical isolation [62]. The panel suggest that structured education should be culturally appropriate and address certain provisions for Aboriginal and Torres Strait Islander peoples. To provide some context, Aboriginal and Torres Strait Islander peoples do not just form one group of people, but there are hundreds of discrete groups; all with distinct languages, social structures, cultural and social traditions, important sites and landmarks, and passing on of traditions, beliefs and customs with storytelling [62]. There should be thought and consultation of whether face-to-face, individual or group approaches would be preferred, and whether educational handouts are culturally appropriate. The inclusion of Aboriginal and Torres Strait Islander artwork and/or flags on educational material may assist in promoting culturally sensitive education. The location of education sessions should also be considered. For example, cultural safety of presenting education “on Country” or in an Aboriginal Community Controlled Organisation. Holding sessions outdoors may also be considered, weather permitting [63].

While more Aboriginal and Torres Strait Islander peoples are progressing through schooling (i.e. achieving national minimum standards for literacy and numeracy), completing year 12, and enrolling in university [58], there may still be reduced health literacy among some Aboriginal and Torres Strait Islander communities. Therefore, foot self-care education should not rely on handouts alone. The panel agreed with the IWGDF that structured education should also account for gender differences and align with the patient’s health literacy and personal circumstances [8]. There must also be consideration of language barriers in consultation, especially where English may be a second, third or fourth language. In these situations, a professional interpreter should be considered.

Health professionals are encouraged to have discussions regarding whether there is regular sharing of shoes and socks within the community. The panel suggests that this should be avoided as to reduce spreading of infections (e.g. fungal infections), and to reduce risk of trauma to the feet related to poor shoe fit or excessively worn footwear. Consideration must be given to the financial cost of footwear, and where possible, more affordable suggestions or recommendations should be made. The panel acknowledge that, in some communities, perhaps many communities, people wear shoes infrequently, or not at all, and this may be for cultural reasons. We recommend health professionals adhere to this prevention guideline wherever possible but may also consider other non-conventional treatment options (e.g. supportive thongs). Health professionals should also have an understanding of Aboriginal and Torres Strait Islander cultural practises (e.g. traditional dance will be performed barefoot to be connected to the land). Perhaps education and consultation with family on how to apply dressings to any cuts or wounds on the sole of the feet prior to cultural activities, and cleaning and redressing any wounds afterwards may be considered.

Most importantly, developing partnerships and engaging with local Aboriginal and Torres Strait Islander health care workers, Liaison Officers and/or community members, such as family and Elders, may assist in promoting these recommendations by determining the best approach for providing education and to ensure it is culturally sensitive. This may optimise understanding and in turn the patient’s outcomes.

E. INSTRUCTIONS ABOUT FOOT SELF-MANAGEMENT

**Q5. In people with diabetes at risk for foot ulceration, is foot self-management compared with no self-management, effective for preventing a first-ever or recurrent DFU?**

### Recommendation 6

Consider instructing a person with diabetes who is at moderate or high risk of foot ulceration (IWGDF risk 2–3) to self-monitor foot skin temperatures once per day to identify any early signs of foot inflammation and help prevent a first or recurrent plantar foot ulcer. The implementation of this recommendation is contingent on validated, user-friendly and affordable systems becoming approved and available in Australia. If the temperature difference is above-threshold between similar regions in the two feet on two consecutive days, instruct the patient to reduce ambulatory activity and consult an adequately trained health care professional for further diagnosis and treatment. (Weak; Moderate).

**Decision:** Adapt.

### Rationale

The panel adapted this recommendation by adding a statement regarding the current lack of availability and approval of this validated, user-friendly technology in Australia (Table 2).

### Summary of justification to adapt

The panel agreed with the IWGDF that the strength of the recommendation is ‘weak’ and the quality of evidence is ‘moderate’ based on the findings from four randomised clinical trials [64–67] and a meta-analysis [52] that support the value of home temperature monitoring and offloading of ‘hot spots’ (i.e. localised areas of inflammation) for the prevention of DFU [8, 52]. The decision not to increase the quality of evidence and strength of recommendation ratings was based on the existing trials having small sample sizes and three of the four trials were conducted in the United States (US); therefore, generalisability outside of the US is unknown. A recent meta-analysis suggested home foot temperature monitoring and reducing of physical activity in response to hot spots halved the risk of foot ulcers in moderate or high risk patients. The significance of findings were however lost in some of the leave one out sensitivity analyses [52].

The panel, including consumer and Aboriginal and Torres Strait Islander representatives, had concerns regarding the acceptability and feasibility of foot temperature monitoring in the Australian context. Currently, there are no validated, user-friendly, and affordable foot skin temperature monitoring devices that have received Therapeutic Goods Administration (TGA) approval in Australia. The TempTouch device (Xilas Medical, San Antonio, TX) was used in all clinical trials [64–67]. It is appropriately calibrated for skin temperatures and is relatively affordable (~$150 USD). Production of this device has however been discontinued and no other validated, user-friendly devices are currently approved or available in Australia. Other infrared dermal thermometers can be purchased in Australia but have not currently been validated for home foot temperature monitoring. For example, DermaTemp (Exergen Corporation, Watertown, MA) is a commonly used device in High-Risk Foot Services (HRFS), however it is not designed for self-monitoring and is significantly more expensive (>$1000 AUD). Another concern with handheld thermometers, like TempTouch, is that the user has to hold the device at 6 different anatomical sites on the sole of each foot and then record and interpret the temperatures at those sites daily [64–67]. This requires substantial time commitment from users and the flexibility to carry out this task daily. At least two more user-friendly foot temperature measuring mats have been designed and are in use in the US [51]. Currently, these are not available in Australia and since they were not included in the randomised trials, it is rather uncertain what effect they may have on ulcer prevention. Hence, the implementation of this recommendation is contingent on validated, user-friendly and affordable systems becoming approved and available in Australia.

While the existing trials [64–67] demonstrated good adherence, it is unclear whether the target Australian population would be accepting of this strategy. To the panel’s knowledge, there has been no evaluation of patient preferences or values for foot temperature monitoring, however, if validated, user-friendly devices become available, people may be willing to devote the extra time to perform the assessment. It is important to note that the existing trials [64–67] demonstrating good adherence may have been affected by selection bias (i.e. participants were likely to have been more motivated individuals).

If it were possible to implement this recommendation in Australia, it would be important to re-evaluate its effectiveness in the real-world setting. For this recommendation to be successfully and equitably implemented in Australia, substantial funding would be required, and as this particular device would be unfamiliar to HRFS clinicians, training would also be necessary. There is also uncertainty regarding the acceptability and use of this device by Aboriginal and Torres Strait Islander peoples. Therefore, further research and consultation with Aboriginal and Torres Strait Islander peoples and health professionals would be needed before home foot temperature monitoring could be recommended to this population.

Despite the above concerns, the panel’s decision to include this recommendation in the Australian prevention guideline was to ensure that clinicians had some guidance for home temperature monitoring should suitable devices be made available in the near future. The panel agreed that home temperature monitoring of the feet could play an important role in the prevention of foot ulceration, and also provide an opportunity for patients to be actively involved in prevention. However, as validated, user-friendly technology is not yet approved or available in Australia, clinicians should be mindful when explaining this intervention and providing advice to their patients.

### Considerations for the Australian context

Foot self-management, which in this case includes monitoring of foot skin temperatures, is a more advanced assistive intervention that requires a person to have ready access to the ability to use an infrared dermal thermometer and be in close communication with an adequately trained health care professional [8]. At the time of writing this guideline, validated, user-friendly and affordable foot skin temperature monitoring devices were not available in Australia. However, should it gain approval for use by the TGA, we suggest the following should be considered by health professionals who are considering using this recommendation: (i) adequate training of health care professionals so that they may educate patients on the use of skin temperature monitoring at home; (ii) assessment of patient ability to perform foot skin temperature monitoring at home (e.g. ability to reach the feet, eyesight, cognition, home environment, support networks, etc); (iii) ensuring that there is adequate support for patients experiencing difficulties in performing the skin temperature monitoring at home or if they have concerns with the results; providing patients with written information and contact details for the health service and/or health professional would be beneficial; (iv) adherence to measuring foot temperatures is an important factor in its effectiveness [65], therefore, this intervention may not suit all patient preferences and lifestyles; (v) people without history of foot ulceration may find daily skin temperature assessments an unnecessary burden [8]; (vi) consideration of costs to the health service and/or patients in procuring this device; (vii) false-positives and false-negatives may unnecessarily concern people and affect their confidence and trust in their assessment results [8, 68, 69]; (viii) there is uncertainty regarding the acceptability for use of this device by the Australian population, including Aboriginal and Torres Strait Islander peoples and those living in rural and remote areas of Australia.

F. ENSURING ROUTINE WEARING OF APPROPRIATE FOOTWEAR

**Q6. In people with diabetes at risk for foot ulceration, is any one specific orthotic intervention, including therapeutic footwear (e.g. shoes, insoles or orthoses) and walking aid, compared with no intervention or another type of orthotic, effective for preventing a first-ever or recurrent DFU?**

### Recommendation 7

Instruct a person with diabetes who is at moderate risk for foot ulceration (IWGDF risk 2) or who has healed from a non-plantar foot ulcer (IWGDF risk 3) to wear medical grade footwear that accommodates the shape of the feet and that fits properly, to reduce plantar pressure and help prevent a foot ulcer. When a foot deformity or a pre-ulcerative sign is present, consider prescribing custom-made footwear, custom-made foot orthoses, or toe orthoses. (Strong; Low).

**Decision:** Adopt.

### Rationale

The panel adopted this recommendation as there was full agreement with the IWGDF regarding the strength of the recommendation, the quality of evidence ratings and its acceptability and applicability in the Australian context (Table 2). The terms ‘therapeutic footwear’ and ‘custom-made insoles’ were replaced with ‘medical grade footwear’ and ‘custom-made foot orthoses’, respectively so that the terminology remained applicable to the Australian context (i.e. would be easier to interpret for an Australian audience) [55].

### Summary of justification to adopt

The panel agreed with the IWGDF that while the quality of evidence for this recommendation is low, the strength of the recommendation is strong [8]. The panel agreed that because people with diabetes at moderate to high risk for DFU (IWGDF risk 2 to 3) have commonly lost the capacity to accurately sense pain or pressure, their ability to judge a tight fit of footwear which may cause damaging tissue trauma, a key risk factor for the development of DFU, is likely to be impaired. We therefore concurred that people at moderate risk of DFU, or those who have healed from a non-plantar DFU, should wear footwear which protects and accommodates the shape of their feet (including adequate length, width, and depth) and focuses on plantar pressure reduction. Footwear should be fitted by appropriately trained professionals, who are able to safely and effectively assess the suitability of a shoe to protect the feet across a whole range of clinical presentations (i.e. mild to severe foot deformity, various gait anomalies), and reduce plantar pressure. Custom-made footwear, custom-made foot orthoses, or toe orthoses should be considered when required to accommodate foot deformity and to help reduce pressure on pre-ulcerative sites or areas prone to tissue trauma. The likelihood of undesirable effects is low; however, the panel acknowledge a degree of wearing in and adjustment is quite often required in the initial period. Visual inspection of the footwear and feet before and after each usage is required during the wear-in period to monitor for any pre-ulcerative signs, injuries or inflammation. Furthermore, routine visual inspection of footwear and orthoses for poor fit or degradation is important. In addition, while for some people footwear selection may be motivated by appearance and/or cost, rather than health-based reasons, the panel agree that avoiding foot ulceration for most people will ultimately override other factors. Put together, the panel agreed this is a strong recommendation when the vital provision of protection from mechanical and thermal trauma was considered alongside the evidence.

### Recommendation 8

Consider prescribing orthotic interventions, such as toe silicone or (semi-)rigid orthotic devices, to help reduce abundant callus in a person with diabetes who is at risk for foot ulceration (IWGDF risk 1–3). (Weak; Low).

**Decision:** Adopt.

### Rationale

The panel adopted this recommendation as there was full agreement with the IWGDF regarding the strength of the recommendation and quality of evidence ratings and its acceptability and applicability in the Australian context (Table 2).

### Summary of justification to adopt

The panel agreed with the IWGDF that the strength of this recommendation is weak, and the quality of evidence is low [8]. Based on the small number of low-quality trials reviewed by the IWGDF, the panel agreed that there may be some small likely desirable effects on preventing a future DFU in considering the use of toe silicone and (semi-) rigid orthoses or felted foam in addition to medical grade footwear, to help reduce abundant callus. The undesirable effects of these interventions are judged to be low in comparison to possible gains, therefore the desirable effects (benefits) of this intervention are deemed to probably outweigh the undesirable effects (risks). There is no published data on patient values regarding these interventions, however, they are frequently used clinically and thus we deem them likely to have reasonable patient acceptability, particularly if they improve comfort, cosmesis and reduce frequency of health care visits for callus reduction.

### Recommendation 9

In a person with diabetes who has a healed plantar foot ulcer (IWGDF risk 3), prescribe medical grade footwear that has a demonstrated plantar pressure relieving effect during walking, to help prevent a recurrent plantar foot ulcer; furthermore, encourage the patient to consistently wear this footwear. (Strong; Moderate).

**Decision:** Adopt.

### Rationale

The panel adopted this recommendation as there was full agreement with the IWGDF regarding the strength of the recommendation and quality of evidence ratings and its acceptability and applicability in the Australian context (Table 2). Similar to recommendation 7, the term ‘therapeutic footwear’ was replaced with ‘medical grade footwear’, so that the terminology remained applicable to the Australian context (i.e. would be easier to interpret for an Australian audience) [55].

### Summary of justification to adopt

The panel agreed with IWGDF that the strength of recommendation was strong and the quality of supporting evidence was moderate [8], as medical grade footwear may reduce the risk of a first-ever foot ulcer in a person at moderate risk for foot ulceration (IWGDF risk 2) [70–72]. As high plantar pressures pose an independent risk factor for the development of DFU [1, 73], and such medical grade footwear [74, 75] has the ability to reduce plantar pressures during walking, its use clinically as a preventative strategy was deemed likely to offer significant benefit. The reduction of elevated plantar pressures at high risk sites in particular, including past ulcer sites and locations of high pressure in the presence of loss of protective sensation, was endorsed. The panel strongly supported the requirement that plantar pressure reduction must be demonstrable (i.e. evidenced) in medical grade footwear prescribed to align with the plantar pressure-guided medical grade footwear protocol adhered to by the RCTs demonstrating a reduction in DFU incidence compared with non-plantar pressure-guided footwear [76, 77]. The panel interpret ‘a demonstrated plantar pressure relieving effect during walking’ as meaning a clinically important reduction in plantar pressure quantified via a valid and reliable, in-shoe plantar pressure measurement system. This may be undertaken, for example, preferably in a footwear prescription/issue consultation in real time by taking plantar pressure measures, or if not possible, to be guided by published, peer-reviewed scientific evidence utilising comparable footwear and/or orthoses. The panel agreed with the IWGDF that ≥ 30% reduction in peak pressure during walking compared with the current (medical grade) footwear, or a reduction of peak pressure to < 200 kPa measured with a validated and calibrated pressure measuring system with sensor size of 2 cm^2^ (updated by IWGDF [8] from past authors referencing 1 cm^2^ [55]) at high-pressure locations should be demonstrated by applying state-of-the-art knowledge of offloading with footwear [8, 55, 76, 77].

Following on, the panel agreed that it is important to encourage consistent wear of the issued footwear whenever weight-bearing, to educate patients on the need for vigilance regarding protection from mechanical trauma. The panel agreed with the IWGDF that the benefits of wearing such medical grade footwear outweigh the risks, while footwear which is an inadequate length or width (i.e. too short or narrow) is likely to increase DFU risk. This emphasises the dual need for sufficient offloading properties and adequate fit. Contrasting preferences around the appearance of footwear are probable undesirable effects of this recommendation for some patients. With the increased availability of visually appealing footwear styles however, and the importance of adequate footwear towards the prevention of chronic and serious DFU, this recommendation is also deemed to be in alignment with what we anticipate a significant proportion of patients’ value. We agreed that cost and availability of both medical grade footwear and pressure measuring technology may limit the applicability of this recommendation in some situations, for example, lack of government funding, however agree that it should remain as an aspirational practice given trials in this area suggest significant ulcer risk reduction may be achievable [8, 78]. Overall, therefore, the panel were closely aligned with all points of the IWGDF on this recommendation.

### Considerations for the Australian context: recommendations 7, 8 and 9

Before prescribing or issuing any offloading device(s), the panel recommends that the benefits, risks and contraindications are always carefully discussed with the patient. It is also important to ensure that patients have an opportunity to discuss and consider their personal circumstances, in order to gain their full informed consent [45, 55]. Providing suitable protection from mechanical trauma in the form of reducing high plantar pressure and/or accommodating foot deformity(s), thereby reducing abundant callus and pre-ulcerative signs, whilst doing no harm, forms a fundamental component of ulcer prevention. Several contextual issues are likely to be pertinent to contemplate in the translation of recommendations 7, 8 and 9 into practice, which individuals should consider in light of their own unique personal situations or circumstances. From the patient perspective, factors such as level and type of physical activity, job requirements and other functional requirements of footwear are likely to come into play. Whilst important for all people with diabetes, these recommendations may be particularly critical for those living in geographically remote areas or where services are limited, as management of mechanical stress using techniques such as medical grade footwear may offer protection from DFU formation. In some, particularly warm places it may be customary to wear footwear such as thongs or slides that are inexpensive, accessible, and easy to put on as compared to closed footwear which is more protective and is fastened to the foot. Alternatively, in other settings, such as in Aboriginal and Torres Strait Islander communities who reside in remote areas, it may be practice to share footwear or to walk barefoot. Clinicians using these prevention guidelines are therefore encouraged to be innovative and flexible in their application to suit the setting, while aiming to uphold the primary principle of minimising damaging trauma as much as possible. To the panel’s knowledge, while there is no evidence that mobility aids may assist in preventing trauma to the feet, this could also be considered following mobility assessment by an adequately trained health professional (if appropriate).

From the perspective of clinical expertise and available resources, there will also be diversity around the country regarding access to suitably trained health professionals at frequent enough intervals, and the availability and funding to pay for devices such as medical grade/custom-made footwear, custom-made foot orthoses, or silicone orthoses. There are a number of funding bodies and equipment schemes available in Australia (e.g. National Disability Insurance Scheme) and clinicians should become familiar with those that apply in their locality. In addition, differing access to validated plantar pressure measurement equipment or measurement services, or lack of high-quality research reporting plantar pressure data in footwear available in these areas, may be a limiting factor. There is also uncertainty regarding the acceptability and utility of the recommended devices for use by Aboriginal and Torres Strait Islander peoples and further consultation with an Aboriginal and Torres Strait Islander health care workers or representatives may be required before they are recommended. In situations where Aboriginal and Torres Strait Islander peoples are not in agreement to use these offloading devices, or prefer a different approach, we suggest consultation and engagement with the patient of what they may consider as more culturally appropriate options. We refer the reader to the Australian Diabetes Footwear Guidelines [55] or the Australian Offloading Guidelines [45] in these circumstances.

In summary, while there will be practical limitations, costs and contextual considerations that must be reconciled for recommendations 7, 8 and 9 to be applied effectively in practice, we agree with the IWGDF that the strong benefits of protection against mechanical and thermal trauma to reducing ulcer risk justifies grading these as strong recommendations. We therefore encourage ongoing and future investment to support the provision of these important recommendations broadly. In regions and settings where these recommendations cannot be applied currently, we suggest continuing to work towards meeting them as aspirational guidelines, particularly as the recommendation for those with a history of DFU are supported by at least moderate quality of evidence, drawing on all locally available innovations and knowledge to meet the guiding principles of minimising damaging trauma.

G. TREATMENT OF RISK FACTORS OR PRE-ULCERATIVE SIGNS ON THE FOOT

**Q7. In people with diabetes at risk for foot ulceration, is treating pre-ulcerative signs on the foot compared with not treating them, effective for preventing a first-ever or recurrent DFU?**

### Recommendation 10

Treat any pre-ulcerative sign or abundant callus on the foot, ingrown toenail, and fungal infection on the foot, to help prevent a foot ulcer in a person with diabetes who is at risk of foot ulceration (IWGDF risk 1–3). (Strong; Low).

**Decision:** Adopt.

### Rationale

The panel adopted this recommendation as there was full agreement with the IWGDF regarding the strength of the recommendation and quality of evidence ratings and its acceptability and applicability in the Australian context (Table 2).

### Summary of justification to adopt

The panel agreed with the IWGDF that the strength of recommendation was strong and the quality of supporting evidence was low [8]. While the panel acknowledged that there was no available evidence supporting the treatment of pre-ulcerative signs, abundant callus on the foot, ingrown toenail, and fungal infection for the prevention of DFU, there was agreement with the IWGDF that the benefit-harm ratio will likely favour the intervention and come at a relatively low cost. We agreed that treatment of presentations including callus, blisters, fissures, ingrown or thickened toenails, cutaneous haemorrhage, and skin and nail fungal infections should be administered by appropriately trained foot care professionals, using current evidence-based methods where available [26] and the level of risk should be considered when selecting treatments and in particular severity of PAD. We refer the reader to the accompanying Australian DFD Guidelines for PAD [40] for further details. The costs associated with this recommendation are likely low and we anticipate that individuals are more likely to value having these minor presentations managed quickly and effectively, over developing secondary, potentially serious, complications such as DFU. Accessibility and applicability of the recommendation is likely to be good. Overall, therefore, the panel were closely aligned with all points of the IWGDF and support this as a strong recommendation.

### Considerations for the Australian context

As the treatments in this recommendation are relatively common and broadly used, there are not many special considerations for the Australian context. As with all recommendations, their application should be evidence-based, where possible, but also locally contextualised [8, 26]. For example, the appropriate treatment of blisters in metropolitan settings, where closed footwear is worn and the weather is cool, may differ compared to rural and remote settings where open shoes are worn, the weather is hot and humid and adhering dressings may be challenging. Options and rationale for management should be fully discussed with the individual and others (i.e. family, carers, traditional healers) as is culturally appropriate, in order to obtain informed consent. We agree with the IWGDF that as these treatments have the potential to lead to harm in people with diabetes if not properly performed, they should only be done by an appropriately trained health care professional (see glossary for definition). Individuals should therefore be educated on how to recognise these issues as part of their home self-checking, and to seek professional treatment if they identify any of these signs rather than trying to treat these issues themselves.

H. SURGICAL INTERVENTIONS

**Q8. In people with diabetes who are at risk of foot ulceration, is performing surgical interventions in comparison to non-surgical intervention, effective for preventing a first-ever or recurrent DFU?**

### Recommendation 11

In a person with diabetes and abundant callus consider digital flexor tendon tenotomy for preventing a first foot ulcer. Where there is an ulcer on the apex or distal part of a non-rigid hammertoe that has failed to heal with evidence-based non-surgical treatment, consider this procedure to help prevent future ulcer recurrence. (Weak; Low).

**Decision:** Adapt.

### Rationale

The panel adapted this recommendation as considered the wording of the original IWGDF recommendation to be confusing, and therefore, restructured the wording to retain the same meaning but improve clarity (see Table 3 for comparative wording).

### Summary of justification to adapt

The panel agreed with the IWGDF that while this recommendation is supported by low quality evidence and is a weak recommendation [8], there may be some situations where digital flexor tenotomy could offer a promising option to help prevent or delay future ulceration [79–85]. For example, in an individual who has pre-ulcerative signs, or an ulcer on a toe where there has been a poor response to evidence-based non-surgical treatment due to a deformity. Risk of ulcer development may also be reduced with flexor tenotomy where there is abundant callus or thickened toenails [81, 83, 85]. Few complications have been reported with flexor tenotomy [79–85], however this finding must be interpreted in light of the low volume and quality of evidence that exists investigating this procedure for the prevention of DFU. Conversely, it is possible that the benefits of flexor tenotomy may outweigh the risks, particularly in people with recurrent ulceration despite best attempts at appropriate, evidence-based, non-surgical intervention.

The panel noted the IWGDF’s perspective that the procedure may be easily performed in an outpatient setting, however questioned availability, costs, and procedural options in the Australian context. It may be that flexor tenotomy is not widely available across Australia, depending on access to health care services and funding models, however the procedure is accessible to some. It was noted that flexor tenotomy should only be performed by appropriately trained, suitably qualified professionals who are able to demonstrate competence in the procedure and registered with the appropriate regulatory body. The panel further agreed with the IWGDF that, taken together, this recommendation is weak.

### Recommendation 12

In a person with diabetes and a plantar forefoot ulcer that has failed to heal with evidence-based non-surgical treatment, consider Achilles tendon lengthening, single or pan metatarsal head resection, metatarsophalangeal joint arthroplasty or osteotomy, to help prevent future ulcer recurrence. (Weak; Low).

**Decision:** Adapt.

### Rationale

The panel adapted this recommendation as considered the wording of the original IWGDF recommendation to be confusing, and therefore, restructured the wording to retain the same meaning but improve clarity (see Table 3 for comparative wording).

### Summary of justification to adapt

Similarly to recommendation 11, while the panel concurred with the IWGDF that the quality of supporting evidence is low and the strength of the recommendation is weak, Achilles tendon lengthening, single or pan metatarsal head resection, and metatarsophalangeal joint arthroplasty may reduce risk of recurrent plantar foot ulceration in some circumstances [86–105]. The panel agreed with the IWGDF, based on their interpretation of the research, that this recommendation applies where a plantar ulcer is not healing in response to evidence-based conservative care, is likely to re-occur due to underlying structural anomalies, has established elevated forefoot plantar pressures, and for Achilles tendon lengthening, ankle joint range of motion is limited, not passing neutral. Importantly though, it is not clear whether the benefits of these procedures outweigh the not inconsequential risks (e.g. new deformities, post-operative infection and transfer ulcers) [89, 105–108], due to the limited quality and quantity of available evidence. Therefore, a clear clinical rationale should be evident before exploring the use of these procedures for DFU prevention, such as to heal a DFU which has not responded to non-invasive evidence-based management and is expected to re-occur if the foot structure is not changed. Further, the surgeon must have adequate training and experience in performing the specific procedure. The panel noted the IWGDF’s point that patient values and preferences for these approaches are unknown and add that this is the case within the Australian context also. It is important therefore that any individuals considering this recommendation fully understand the range of risks and benefits in order for them to personally gauge acceptability for their unique situation and health care preferences. It was noted that these surgical procedures should only be performed by appropriately trained, suitably qualified professionals who are able to demonstrate competence in the procedure and registered with the appropriate regulatory body. The panel further agreed with the IWGDF that this is a weak recommendation.

### Recommendation 13

We suggest not to use a nerve decompression procedure, in preference to accepted standards of good quality care, to help prevent a foot ulcer in a person with diabetes who is at moderate or high risk of foot ulceration (IWGDF risk 2–3) and who is experiencing neuropathic pain. (Weak; Low).

**Decision:** Adopt.

### Rationale

The panel adopted this recommendation as there was full agreement with the IWGDF regarding the strength of the recommendation and quality of evidence ratings and its acceptability and applicability in the Australian context (Table 1).

### Summary of justification to adopt

Unrecognised nerve entrapment may coexist in patients with diabetes-related sensorimotor peripheral neuropathy [109]. While nerve decompression procedures have demonstrated low incidence rates for new and recurrent ulcers in observational studies with prolonged follow-up periods [109–113], there is no available high-quality evidence from controlled studies or trials that this procedure has an ulcer prevention effect [8]. Although this type of procedure may be considered in certain clinical scenarios, the panel agreed with the IWGDF that the strength of the recommendation is weak and the quality of the evidence is low, particularly as there have been no studies that have compared nerve decompression to standards of good quality care [8]. Similar to recommendations 11 and 12, it is also not clear whether the potential benefits of this particular procedure outweigh the not inconsequential risks of such a surgical procedure (e.g. post-operative infection, delayed wound healing, permanent nerve damage) due to the limited quality and quantity of available evidence. The panel agreed with the IWGDF that the balance of effects most likely favoured good quality care, rather than the surgical intervention, particularly considering the acceptability and feasibility (e.g. cost and inconvenience) of the intervention. Patient values and preferences for this surgical approach are also unknown due to a lack of evidence, and this is also the case within the Australian context. On all other points of assessment for recommendation 13, the panel were closely aligned to the rationale of the IWGDF.

### Considerations for the Australian context: recommendations 11, 12 and 13

For any surgical intervention it is of upmost importance that an individual be fully informed about what the procedure involves including the likely benefits (desirable effects) versus risks (undesirable effects) compared to good quality of care or other treatment options, to support their autonomy and informed decision making. Full disclosure is important for surgical procedures given it is a permanent, invasive intervention which may have important physical but also psychosocial implications for some individuals (e.g. managing expectations, illness anxiety, potential for reduced functional capacity, ability to work, etc). Health professionals should very carefully assess, and discuss with the patient, adverse events in the context of their unique situation and the procedure(s) being considered, in particular where there is PAD (please see the accompanying Australian DFD Guidelines for PAD [40]), increased potential for non-healing of the wound/surgical site or any other situation which may result in poor post-operative outcomes. Access to these procedures may be limited in the Australian context, depending on the level of surgical intervention available at local health services. We suggest that when discussing the above benefits, risks, contraindications, and personal circumstances for these surgical procedures with people living in rural and remote areas of Australia, that they should be carefully considered in light of potential for limited access to follow-up appointments for close monitoring of progress and for potential complications. It is likely that these people would need to travel to large metropolitan tertiary hospitals to receive these procedures and post-operative care; all of which should be discussed as part of the informed consent process. In addition to the above, similar considerations for Aboriginal and Torres Strait Islander peoples apply. All discussions with Aboriginal and Torres Strait Islander peoples should be preferably performed in conjunction with family and/or an Aboriginal and Torres Strait Islander health care worker. It is also important to allow adequate time to discuss, understand and consider the benefits, risks, contraindications, personal circumstances, and travel requirements of such procedures; to enable the person and their family to make an informed decision. To the panel’s knowledge, we are unaware of any guidelines that focus on culturally appropriate discussions surrounding surgery with Aboriginal and Torres Strait Islander peoples. The development of such guidelines would be most useful. Otherwise, for considerations of these same surgical procedures for people with foot ulcers, please refer to the accompanying Australian DFD Guidelines for offloading treatment [45].

I. FOOT-RELATED EXERCISES AND WEIGHT-BEARING ACTIVITY

**Q9. In people with diabetes at risk for foot ulceration, are foot-related exercises compared with no foot-related exercises effective for preventing a first-ever or recurrent DFU?**

### Original IWGDF recommendation

Consider advising a person with diabetes who is at low or moderate risk for foot ulceration (IWGDF risk 1 or 2) to perform foot and mobility-related exercises with the aim of reducing risk factors of ulceration, that is, decreasing peak pressure and increasing foot and ankle range of motion, and with the aim of improving neuropathy symptoms. (Weak; Moderate).

**Decision:** Excluded.

### Rationale

The panel excluded this recommendation based on having substantially differing judgements to the IWGDF for desirable effects, balance of effects and the quality of supporting evidence, resulting in the panel concluding that the recommendation should be excluded as high quality clinical trials specifically investigating the effectiveness of foot and mobility-related exercises on ulcer prevention are non-existent (Table 1) [8].

### Summary of justification to exclude

Exercise is widely accepted as being valuable for maintaining or improving physical and mental health in the general population [114]. There is also good evidence that exercise training can improve balance and gait, reduce falls risk and improve other functional markers in people with diabetes at risk of foot ulceration [115]. While there is some evidence to suggest that foot and mobility-related exercises may improve modifiable risk factors for foot ulceration, such as plantar pressures, foot and ankle joint range of motion and neuropathy symptoms [116–125], there is no current convincing evidence that foot and mobility exercises have a *preventative effect* on foot ulceration. Therefore, the panel disagreed with the IWGDF regarding the inclusion of this recommendation in the Australian prevention guidelines [8].

The panel’s decision to exclude this recommendation from the Australian prevention guideline was based on the following: (i) recommendation is based on very low quality of supporting evidence (small studies with inconsistent findings for surrogate outcomes only); (ii) our judgement of “don’t know” based on the absence of direct evidence for the desirable effect (benefit) of foot and mobility-related exercises for the prevention of foot ulceration is non-existent; (iii) foot and mobility-related exercises are likely to be time consuming, adherence challenging, and patients may already feel overwhelmed by their general diabetes management. Therefore, advising patients to routinely perform foot and mobility-related exercises without any evidence of clinically important benefit, may be considered inappropriate and an unnecessary burden on the patient. The panel were also unclear on what the patient values and preferences would be for performing these exercises, as this was ambiguous in existing studies. Although some individuals are not keen to do exercises overall, there is no reason to suspect that exercises would not be acceptable or valuable to individuals. Adverse events related to performing such exercises are also unknown, however, the panel suggested that any new weight-bearing exercise regime could increase the risk of ulceration, particularly if there was a sudden increase in activity. In cases where foot or mobility-related exercises are indicated (e.g. for treatment of a musculoskeletal pathology), the panel recommends that patients undergo a thorough foot assessment to establish risk of ulceration by a trained health care professional prior to prescribing an exercise program. The panel agreed with the IWGDF that prescribed foot-related exercises that mechanically load the foot are contraindicated in people with pre-ulcerative signs or an active foot ulcer [8].

### Considerations for the Australian context

Although it is currently unknown, there is no reason to expect that foot and mobility-related exercises would not be acceptable to the overall Australian population. However, as mentioned previously, as there is no direct evidence for the benefit of these exercises on the prevention of foot ulceration, the panel does not recommend the prescription of foot and mobility-related exercises for the purpose of foot ulcer prevention within Australia. Therefore, the panel excluded this recommendation from the Australian prevention guideline.

**Q10. In people with diabetes who are at risk for foot ulceration, can the level of weight-bearing daily activities be safely increased without increasing first-ever or recurrent DFU risk?**

### Recommendation 14

Consider communicating to a person with diabetes who is at risk of foot ulceration (IWGDF risk 1–3) that any increase in weight-bearing activity should be gradual, ensuring appropriate footwear and/or prescribed offloading device(s) are worn, and that the skin is frequently monitored for pre-ulcerative signs or injury. (Weak; Low).

**Decision:** Adapt.

### Rationale

The panel adapted this recommendation by restructuring the IWGDF wording to focus on a gradual increase in weight-bearing activity as the panel considered there was limited evidence to specifically prescribe moderate increase in the level of walking-related weight-bearing activity (i.e. an extra 1000 steps/day), but broadly considered a gradual increase under certain conditions (such as when appropriate footwear and/or prescribed offloading device(s) for the patient were worn during the activity) could be supported. Thus, the wording was also amended to improve clarity of the recommendation (Table 3).

### Summary justification to adapt

Exercise is known to have important benefits for cardiovascular and metabolic health [114, 126, 127]. However, the ideal approach to increasing exercise, particularly with respect to weight-bearing, is unclear. It makes logical sense that any attempt to increase exercise should be graduated, preferencing activity that limits plantar pressure and shear on the feet. Activities involving limited weight-bearing, such as recumbent bike or pool-based exercise, may be considered where suitable for the individual. Additional precautions, based on expert opinion [8], such as checking skin integrity before and after any exercise, wearing of socks and well-fitting/appropriate footwear, and regular podiatry review may help to reduce risk. While the panel agreed with the IWGDF that the quality of evidence is low and the strength of the recommendation is weak, the panel disagreed with the IWGDF, based on our interpretation of the research, that there is sufficient evidence to provide a specific recommendation on the level of weight-bearing activity increase that is likely to be safe or harmful [8]. Of the two RCTs [127, 128] evaluating the effects of weight-bearing exercise in people with diabetes and peripheral neuropathy, the study populations were small in size (*n* = 79 and *n* = 29, respectively) and with relatively short periods of follow-up (12 months and 12 weeks, respectively). It seems reasonable to suggest that any increase in physical activity should be gradual, however it is the expert opinion of the panel that it is not possible to advise on precise step figures based on the existing evidence. Generally, gradual increases in weight-bearing activity would most likely be acceptable and feasible to patients and providers within Australia, including Aboriginal and Torres Strait Islander peoples. However, with respect to the values, this would vary, and care should be taken to explain what gradual increase of exercise would be for each individual.

Exclusion of this recommendation was initially considered by the panel due to a lack of empirical evidence, however, the panel believed that some expert guidance around weight-bearing physical activity would be of great clinical importance for two main reasons. First, there is sound evidence regarding the benefit of physical activity in the general population and in people with diabetes, particularly with respect to cardiovascular benefit [114, 126]. Second, some guidance to patients planning to increase physical activity may help to prevent avoidable ulceration; as rapidly increased weight-bearing may result in cumulative plantar tissue stress [129]. Therefore, the panel decided to adapt this recommendation based on the above rationale.

### Considerations for the Australian context

All things considered, people with diabetes at-risk of foot ulceration should not be discouraged from carefully increasing exercise. This is based on the potential health benefits and evidence being equivocal as to whether weight-bearing activity exposes the individual to any greater ulceration risk [130]. The relative importance of functional outcomes, potential cardiovascular/metabolic benefits, and risk of injury (both ulceration and musculoskeletal) would vary on an individual level. Therefore, this recommendation should be individualised and implemented according to the person’s unique situation. For example, in those at high risk of foot ulceration, activities involving limited weight-bearing (such as recumbent bike or pool-based exercise) may be considered more appropriate.

Considerations on how to implement this recommendation would depend on: (i) appropriate advice being given to persons with diabetes by an adequately trained health care professional, including a prior foot assessment to establish risk status and monitoring of skin for pre-ulcerative signs; (ii) appropriate support of individuals attempting to increase weight-bearing activity (e.g. exercise physiology, rapid access to advice in case of injury); and (iii) appropriate equipment, especially footwear, being available with particular reference to reasonable options for Aboriginal and Torres Strait Islander peoples. When weight-bearing activities are performed in geographically rural or remote areas of Australia with hot climates, this may precipitate excess perspiration and increased risk of blistering and/or ulceration in patients residing in these areas. Therefore, these individuals would most likely benefit from more regular monitoring of their feet from a health professional, for the early identification and management of pre-ulcerative signs and injuries. Telehealth services may assist in these regions of Australia, and particularly where there is limited scope for patients to attend face-to-face appointments for advice on gradual increase in activity, appropriate footwear and how to monitor the feet for pre-ulcerative lesions [51, 57]. The panel suggests that any advice on increasing weight-bearing activity provided to Aboriginal and Torres Strait Islander peoples should be performed in collaboration with local Aboriginal and Torres Strait Islander health care workers and/or with input from family and Elders.

J. INTEGRATED FOOT CARE

**Q11. In people with diabetes at risk for foot ulceration, is providing integrated foot care compared with not providing integrated foot care, effective for preventing a first-ever or recurrent DFU?**

### Recommendation 15

Provide integrated foot care for a person with diabetes who is at high risk of foot ulceration (IWGDF risk 3) to help prevent a recurrent foot ulcer. This integrated foot care includes professional foot care, adequate footwear, and structured education about self-care. Repeat this foot care or re-evaluate the need for it once every one to 3 months, as necessary. (Strong; Low).

**Decision:** Adopt.

### Rationale

The panel adopted this recommendation as there was full agreement with the IWGDF regarding the strength of the recommendation and quality of evidence ratings and its acceptability and applicability in the Australian context (Table 2).

### Summary of justification to adopt

The panel agreed with the IWGDF that there is a low quality of supporting evidence for this recommendation [8]. However, given that the provision of integrated foot care provides an opportunity for screening of the feet for any pre-ulcerative signs or problems, early intervention, and an opportunity for foot health counselling and education, all of which are earlier individual recommendations for prevention (see Recommendations 3,4,5,10 and 14), the panel supports the IWGDF’s ‘strong’ recommendation rating [8]. This was based on the panel agreeing with the IWGDF that the combined benefits of these recommendations are likely to increase the magnitude of the overall desirable effects on DFU prevention and hence the balance of effects are likely to clearly favour the integrated foot care intervention over standard care. While there may be some perceived inconvenience for some patients to attend a health service for regular foot care (particularly for those with multiple health care needs and appointments), the panel suggests that the desirable effects (benefits) outweigh any potential undesirable effects of harms or burden to the patient. Otherwise, the panel concluded that the intervention is applicable, affordable for most patients, and the resources and expertise are available via most organisations that would provide such integrated foot care in Australia. On all other points of assessment for recommendation 15, the panel were closely aligned to the rationale of the IWGDF.

### Considerations for the Australian context

Similar to the considerations for the Australian context outlined in recommendation 4, integrated foot care is particularly important for those living in geographically rural and remote areas of Australia with hot climates that may precipitate perspiration and increased risk of blistering and/or ulceration. Due to the often dry and dusty environments in rural and remote regions of Australia, patients residing in these areas would most likely benefit from regular monitoring of their feet from a health professional for the early identification and management of injuries, such as abrasions from foreign objects, or pre-ulcerative signs. Telehealth services may assist in these regions of Australia, and particularly where there is limited scope for patients to attend face-to-face appointments.

Similar to people living in geographically remote locations, the panel also considers that this recommendation is particularly important for Aboriginal and Torres Strait Islander peoples, given the increased risk of foot ulceration and access to medical care may not be as frequent. As per recommendation 4, the panel suggests that structured education (a component of integrated foot care) should be performed in collaboration with local Aboriginal and Torres Strait Islander health care workers and/or with input from family and Elders to optimise understanding and individual outcomes. It may also be beneficial for service providers to promote consistency of staff who are providing integrated foot care to Aboriginal and Torres Strait Islander peoples; as this may assist in expedited rapport building and trust between the person and health professional, which may result in a more enriched clinical experience for the patient. Telehealth services for the structured education component of integrated foot care may also be considered for Aboriginal and Torres Strait Islander peoples, however, the views and experiences, acceptability and appropriateness of using telehealth services in this population requires further investigation [131].

## Discussion

### Key findings and recommendations

This is a long overdue new Australian guideline on prevention of DFU, produced by systematically adapting all IWGDF prevention recommendations to the Australian context. Overall, we adopted nine, adapted six and excluded one of the 16 original IWGDF recommendations. For the prevention of DFU in Australia, the panel recommends the following: (i) screening all people with diabetes at increased risk of foot ulceration at intervals corresponding to the IWGDF risk ratings; (ii) providing structured education about foot protection, inspection, footwear, weight-bearing activities and foot self-care (and potentially self-monitoring of foot skin temperatures contingent on device approval and availability in Australia); (iii) prescription of orthotic interventions and/or medical grade footwear; (iv) providing integrated foot care. If the above recommended non-surgical treatment fails, the use of various surgical interventions for the prevention of DFU can be considered. This guideline should serve as the new national evidence-based prevention guideline and the best practice standard for implementing prevention strategies in people at-risk for DFU in Australia.

### Clinical implementation and considerations for the Australian context

To optimise and promote the uptake of these new prevention recommendations into national clinical practice, we provided a comprehensive range of implementation considerations for health professionals, and for the first time, have included considerations for people residing in geographically remote areas of Australia and people who identify as an Aboriginal and Torres Strait Islander person. In addition, all prevention recommendations were incorporated into a one-page user-friendly clinical pathway to try and maximise uptake and implementation of these recommendations and considerations by busy multi-disciplinary clinicians in Australia (Fig. 2).

Before implementing any of the prevention recommendations outlined in this guideline, the panel suggests that health professionals consider them in light of their health service policies and resources, clinical expertise, and the needs of their individual clients. Given these recommendations have been assessed and written in accordance with the Australian context, the implementation should be applicable and feasible to most health service providers across the country. However, prior to the implementation of these guidelines, it is important for health professionals to understand and reflect on the health disparities that still exist between geographically remote and metropolitan populations and importantly between Aboriginal and Torres Strait Islander peoples and non-Indigenous Australians [59–61, 132]. These health disparities are most likely associated with government policy and a complex historical legacy of social determinants of health affecting the Aboriginal and Torres Strait Islander population [131, 132]. Further, there are still challenges that exist in the delivery of effective, equitable, culturally sensitive and responsive health care for this group of individuals [131, 132]. This is particularly evident within rural and remote regions of Australia, where Aboriginal and Torres Strait Islander people account for a higher proportion of the population [62, 131]. With all of these factors combined, it is not unexpected that there is higher incidence of DFU and poorer outcomes observed in Aboriginal and Torres Strait Islander populations [5, 13].

Health services and clinicians should continue to strive for effective, equitable and culturally appropriate clinical environments to all Australians at-risk of DFU, but particularly for those most vulnerable such as Aboriginal and Torres Strait Islander peoples and those living in rural and remote regions of Australia. As mentioned previously, Aboriginal and Torres Strait Islander peoples are from numerous discrete groups [62]. Therefore, an important first step for health professionals is to determine the best approach to provide culturally sensitive education and treatment, and how best to meet the needs of their patients [133]. One way to achieve this, is to work in partnership and to foster meaningful relationships with representatives from the community, for example, Aboriginal and Torres Strait Islander health care workers, family members and/or Elders. Providing culturally responsive health care through the provision of a safe and welcoming clinical environment that is professional, humble, inclusive, transparent, respectful, empathetic, non-judgemental, and that gives a ‘voice’ which encourages client choice and informed consent, may result in improved health outcomes in the Aboriginal and Torres Strait Islander population [133]. Structured education should also account for gender differences and align with the patient’s health literacy, preferences and values, and personal circumstances [8]. Finally, due to potential limited access, movement (e.g. cultural practices), greater severity of diabetes and risk of complications of some Aboriginal and Torres Strait Islander peoples, health professionals may also consider opportunistic screening and/or more frequent screening intervals.

Special considerations should also be made for the delivery of these prevention recommendations for those residing in rural or remote areas of Australia. New national initiatives in the use of telehealth, multi-media platforms and/or HRFS teams visiting communities may prove to be invaluable in improving health access and equity for these individuals, and overall health outcomes. Equipment and resources for this approach would need to be made available to the health care services and patients, which may not always be feasible in some locations. Resourcing of outreach programs in association with secondary and tertiary health care organisations may be a tangible and effective solution.

### Limitations

While the recommendations presented in this Australian prevention guideline were adapted from the high-quality IWGDF prevention guideline [8], the panel followed a robust protocol to systematically assess each of the 16 IWGDF recommendations for their quality of evidence, strength of recommendation and acceptability and applicability to the Australian context [29]. Although updated systematic reviews were not performed since the 2019 IWGDF systematic reviews and no new systematic reviews for different questions were available in this process, it is unlikely that new evidence would have substantially impacted the Australian recommendations; particularly as the IWGDF prevention guidelines were published so recently [8]. As a measure to ensure all relevant literature was included in the current Australian guideline, any Australian literature related to the prevention of DFU published after the IWGDF guideline, was eligible for inclusion [29]. For the most part, despite the widespread clinical application of prevention interventions, the empirical evidence underlying these recommendations is lacking and is often based on expert opinion. In Australia, there is particularly limited research on the prevention of DFU, and research within the Aboriginal and Torres Strait Islander population is essentially non-existent. The limited available evidence does not imply that these prevention interventions are not effective for Australians, but rather more research is required to provide a stronger evidence base. The clinical implementation of the recommendations outlined in this prevention guideline also requires further investigation, particularly encompassing Aboriginal and Torres Strait Islander peoples and those living in rural and remote regions of Australia. Finally, as the recommendations within this guideline centre around the IWGDF risk stratification ratings, this may limit the scope and applicability of the guideline in terms of catering for all individual needs and personal circumstances. For example, the guideline may not always provide the right treatment, for the right person, at the right time [8].

### Strengths

This new Australian evidence-based prevention guideline used a rigorous methodological approach to systematically assess all of the recommendations outlined in the IWGDF prevention guideline and outlined considerations specific to the Australian context [29]. The panel consisted of a multi-disciplinary team of (inter) national experts in the field of preventing DFU including Vascular Surgery, Endocrinology, Podiatry and Pedorthics. A point of difference to the IWGDF, was that the Australian panel also included consumer representatives (patient with history of DFU and an Aboriginal and Torres Strait Islander person/health care worker). This was to ensure that the Australian guideline was pragmatic, clinically relevant, considered patient values and preferences, and was applicable to the Australian context. Another strength specifically related to the Australian guideline, was that an evidence-based clinical pathway was developed for health professionals in order to assist implementation and accessibility of the recommendations.

We anticipate that the broad implementation of these guidelines throughout Australia will lead to improved health outcomes in those at-risk of DFU. There are several benefits for the use of this new guideline in clinical practice. First, it should encourage evidence-based consistency of care among health services and health professionals, which may in turn improve clinical pathways of care and reduce any confusion for health professionals and their patients at-risk of DFU. Second, it should help guide and give confidence to clinicians providing evidence-based DFU prevention strategies. Third, as the guideline has been designed to be evidence-based, yet pragmatic, it is likely that these best practice recommendations can be implemented by all health professionals involved in DFU prevention in Australia, providing that they are adequately trained. Finally, health professionals following these recommendations should achieve better prevention and overall outcomes for their patients with DFU in Australia.

### Future research directions summary

The panel acknowledges that prevention of DFU is under-studied and there is a need to improve the evidence-base in this area. The panel identified the following future research priorities:

1. The effectiveness of screening for ulcer prevention, including what factors to screen for, validity of screening tools/techniques and combinations, and optimal duration matched to patient presentation (physical, psychological, social), assessment intervals, etc.
2. Well-designed trials for preventative surgical procedures with longer follow-up periods
3. Well-designed trials to investigate whether foot and mobility-related exercises reduce DFU incidence/modify risk factors (e.g. plantar pressure) and which types and combinations of exercise are most effective
4. User-friendly, accessible, accurate, reliable, and cost effective methods to monitor foot temperatures at home, and evaluation of patient preferences or values
5. The associated costs and cost effectiveness of prevention interventions at both an individual and societal level compared with usual care
6. Integrated foot care approach combining all the recommendations outlined in this guideline on preventing DFU
7. Research on the prevention of DFU within the Aboriginal and Torres Strait Islander population broadly
8. Structured education approaches within the Aboriginal and Torres Strait Islander population and those in rural and remote areas, including use of Telehealth services, etc.
9. Psychological interventions to support adherence and psychosocial management in relation to DFU prevention
10. Optimal medication management on prevention of DFU
11. More effective ways to implement preventative care
12. Risk and benefit of exercise programs typically recommended to improve cardiovascular health in people at risk of DFU, and whether specific modalities minimise risk of ulceration (e.g. walking, bike, rowing, swimming)

## Conclusion

The effective prevention of DFU is critical to reducing this serious and costly health problem. These new Australian guidelines provide evidence-based recommendations for DFU prevention and have been developed to suit the needs of consumers and health professionals in the context of the unique geography, diversity, cultures, and health care settings in Australia. This guideline includes specific considerations and simplified clinical pathways for Australian health professionals to follow, which may help to optimise implementation of these prevention recommendations in clinical practice. Health professionals following these recommendations should achieve better DFU prevention outcomes and help to reduce the large national burden of DFU in Australia.

## Data Availability

Data sharing is not applicable to this article as no datasets containing patient information were generated or analysed during the current study.

## Abbreviations

ADS: Australian Diabetes Society
DFA: Diabetes Feet Australia
DFD: Diabetes-related foot disease
DFU: Diabetes-related foot ulceration
ESRD: End-stage renal disease
EtD: Evidence to Decision
GRADE: Grading of Recommendations, Assessment, Development and Evaluations
HRFS: High-risk foot service
IWGDF: International Working Group on the Diabetic Foot
LOPS: Loss of protective sensation
NHMRC: National Health and Medical Research Council
PAD: Peripheral artery disease
RCT: Randomised controlled trial
TGA: Therapeutic Goods Administration
US: United States

## Glossary*

Abundant callus: Callus that, as assessed by an appropriately trained health care professional, requires debridement to reduce risk for ulceration.
Adequately trained health care professional: A person who according to national or regional standards has the knowledge, expertise, and skills to perform a specified task in screening, examining, or managing a person with diabetes who is at risk of foot ulceration.
Adherence: The extent to which a person’s behaviour corresponds with agreed recommendations for treatment from a health care provider, expressed as quantitatively as possible; eg, the proportion of time, steps or instances that the prescribed intervention (or comparator) is used [134].
Custom-made (medical grade) footwear: Footwear uniquely manufactured for one person, when this person cannot be safely accommodated in pre-fabricated (medical grade) footwear. It is made to accommodate deformity and relieve pressure over at-risk sites on the plantar and dorsal surfaces of the foot. In-depth assessment, multiple measurements, impressions or a mould, and a positive model of a person’s foot and ankle are generally required for manufacture. This footwear includes a custom-made orthotic and may include other orthotic components. Also known as “bespoke footwear”, “therapeutic footwear” or “orthopaedic footwear”.
Custom-made foot orthoses: An insole that is custom-made to the individual’s foot using a 2D or 3D impression of the foot, and that is often built-up in a multi-layer construction. This may also incorporate other features, such as a metatarsal pad or metatarsal bar. The orthotic is designed to conform to the shape of the foot, providing cushioning and redistribution of plantar pressure. The term "orthotic" is also known as "insert" or "liner" or "insole".
Extra-depth footwear: Pre-fabricated footwear constructed with additional depth and volume in order to accommodate deformity such as claw/hammer toes and/or to allow for space for a thick orthotic. Usually a minimum of 5 mm (~ 3/1600) depth is added compared with off-the-shelf footwear. Even greater depth is sometimes provided in footwear that is referred to as double depth or super extra-depth.
Foot deformity: See IWGDF definitions and criteria document [135].
Foot-related exercises: Any physical exercise specifically targeting the foot or lower-extremity with the aim of changing foot function. These exercises can include stretching and strengthening of the foot and ankle musculature and functional exercises such as balance and gait training. These exercises are provided and/or supervised by a physical therapist or a similarly adequately trained health care professional.
Foot self-care: Foot care interventions the patient can do at home, consisting of but not limited to: foot inspection, washing of feet, careful drying between the toes, nail cutting, using emollients to lubricate skin, footwear inspection, avoidance of using chemical agents or plasters to remove callus, avoidance of walking barefoot or on socks only or in thin-soled slippers, avoidance of wearing tight socks, and avoiding exposure to excessive cold and heat.
Foot self-management: Advanced assistive interventions the patient can use at home, consisting of but not limited to: home monitoring systems, lifestyle interventions, telehealth, technological applications, peer support programs.
Footwear: Defined broadly as any shoe-gear and including insoles.
Footwear modification: Modification to existing footwear with an intended therapeutic effect, for example, pressure relief.
Hosiery: Stockings or socks of any kind. See further Stockings or Socks.
In-shoe (semi-)rigid orthosis: Term used for device put inside the shoe to achieve pressure reduction or alteration in the function of the foot. Can be pre-fabricated or custom-made.
Limited joint mobility: See IWGDF definitions and criteria document [135].
Medical grade footwear: Footwear that meets the specific needs of a person. Can be either pre-fabricated (see “Pre-fabricated medical grade footwear”) or custom-made (see “Custom-made medical grade footwear”). Also known as pedorthic footwear.
Off-the-shelf footwear: Readily available footwear that has not been modified and has no intended therapeutic functions. Preferred term is pre-fabricated footwear.
Pre-fabricated insole: An “off-the-shelf” flat or contoured insole made without reference to the shape of the individual patient’s foot.
Pre-fabricated medical grade footwear: Pre-fabricated footwear that meets the specific needs of a person, on the basis of footwear that provides extra depth, multiple width fittings and features designed to accommodate a broader range of foot types. Other features may include modified soles, fastenings and smooth internal linings. This type of footwear is usually available at specialty shoe shops.
Shoe last: Last used to make footwear. The upper of the footwear is moulded or pulled over the last. The last shape defines the footwear shape including the outsole, heel pitch and toe spring. For off-the-shelf or pre-fabricated footwear generically generated lasts in different sizes are used.
Slipper: Low-cut, open type footwear that is easily slipped onto the foot. Includes thin-soled slippers and flip-flops (thongs).
Socks: Garment for the foot and lower part of the leg, typically knitted from wool, cotton, or nylon.
Stockings: Garment that fits closely over the foot and lower leg, typically elastic. Includes compression stockings for medical purposes.
Structured education: Any educational modality that is provided in a structured way. This can take many forms, such as one-to-one verbal education, motivational interviewing, educational group sessions, video education, booklets, software, quizzes, and pictorial education via animated drawing or descriptive images.
Therapeutic footwear: Generic term for footwear designed to have some therapeutic effect that cannot be provided by or in off-the-shelf footwear. Custom-made shoes or sandals, custom-made foot orthoses, extra-depth shoes, and custom-made or prefabricated medical grade footwear are examples of therapeutic footwear.
Toe orthosis: An in-shoe orthosis to achieve some alteration in the function of the toe.
Weight-bearing activity: Activity during which the foot is loaded by supporting the body weight of the person, and expressed as quantitatively as possible. Includes walking and standing.
